# Assessment of psychological problems in children with chronic physical health conditions using DSM-oriented ASEBA scales: a meta-analysis

**DOI:** 10.3389/fpsyg.2026.1930737

**Published:** 2026-10-16

**Authors:** Martin Pinquart, Lea Lütke-Lengerich

**Affiliations:** Department of Psychology, Marburg University, Marburg, Germany

**Keywords:** Achenbach system of empirically based assessment, child behavior checklist, chronic illness, chronic physical health conditions, meta-analysis

## Abstract

**Background:**

Chronic physical health conditions (CPHCs) increase the risk of mental health problems. The present meta-analysis integrates research of studies that compared DSM-oriented scores from the Achenbach System of Empirically Based Assessment (ASEBA) in young people with CPHCs and a control condition.

**Methods:**

A systematic search in electronic databases (PsycInfo, Medline, Google Scholar, and PSYNDEX) identified 115 studies involving 12,958 children and adolescents with CPHCs and 24,638 control group members which were included in a hierarchical meta-analysis.

**Results:**

Elevated scores were found on all DSM-oriented scales, with the highest effect sizes observed for Somatic Problems (*g* = 0.66 standard deviation units, confidence interval [CI] 0.56 to 0.75) and Affective Problems (*g* = 0.61, CI 0.53 to 0.69). Between 8.6% (Attention Deficit/Hyperactivity Problems) and 14.3% (Affective Problems) of the young people with CPHCs scored in the clinical range. Effect sizes were larger if parent ratings rather than adolescent or teacher ratings were used, in the case of direct neurological involvement and of shorter duration of the CPHCs, in older samples, and when test norms rather than healthy comparison groups were used.

**Conclusion:**

We conclude that the ASEBA is a recommended screening tool for children with CPHCs but more direct comparisons of results from the DSM-based scales and the empirically derived ASEBA scales are needed to inform the choice of the scales.

**Systematic review registration:**

https://osf.io/nfvdj.

## 1 Introduction

Chronic physical health conditions (CPHCs) are often conceptualized as physical conditions that last for more than 3 months (Bernell and Howard, 2016). CPHCs have been found to be associated with higher psychological problems in children and adolescents (Pinquart, 2024, 2026), thus indicating the need for screening.

Although a comprehensive formal model of the relationship between CPHCs and psychological symptoms is not yet available, the following factors have been described as potential sources of elevated psychological symptoms: restrictions in positive physical and social activities due to functional impairments or hospital stays, loss of control, social stigma, executive dysfunction, risks for further deterioration, and symptom overlap between CPHCs and psychological symptoms (e.g., Pinquart, 2024). While potential symptom overlap plays a role in most CPHCs, physical restrictions and restricted control over one’s body are particularly relevant in neurological and neuromuscular disorders (Whelan and Walker, 2009). Negative reactions by others play an important role in cases of visible CPHCs (Pinquart, 2017). Finally, executive dysfunction is prevalent in some neurological CPHCs (van den Berg et al., 2021). Thus, when screening for psychological problems across a broad range of CPHCs, somatic problems are expected to be the most prevalent (due to symptom overlap with many CPHCs), while externalizing problems associated with impaired executive function are expected to be the least prevalent (due to their restriction to a narrow range of neurological CPHCs). Previous meta-analyses using different screening tools have found this to be the case (Pinquart, 2024, 2026).

The Achenbach System of Empirically Based Assessment (ASEBA; Achenbach and Edelbrock, 1983; Achenbach and Rescorla, 2001) is probably the most frequently used instrument for screening these symptoms in children and adolescents (Pinquart, 2026). Originally, it consisted of eight empirically derived symptom scales, such as anxious/depressed or withdrawn/depressed for six-to-eighteen-year-olds and six to seven scales for younger children, depending on whether ratings from parents or formal caregivers are used. These scales were developed based on a series of factor analyses of items rated by parents, teachers, or adolescents in clinical and nonclinical samples. Parallel versions for parents, teachers/formal caregivers, and adolescents are available.

In addition to the empirically derived scales, six DSM-oriented scales were introduced in 2001. These scales consist of ASEBA items that experts from 16 cultures identified as being highly consistent with DSM-IV diagnostic categories. These scales address Affective Problems, Anxiety Problems, Somatic Problems, Attention Deficit Hyperactivity (ADH) Problems, Oppositional Defiant Problems, and Conduct Problems (Achenbach and Rescorla, 2001; Achenbach, 2013). However, these scales are not direct counterparts of all DSM diagnostic criteria. For example, they do not include information on symptom duration that would be needed for clinical diagnosis. When relating diagnoses with clinical interviews, the DSM-based ASEBA scales exhibit greater specificity than the empirically derived scales (Bellina et al., 2013). Nonetheless, some studies found poor concurrent validity with a gold standard clinical interview (Chromik and Friedman, 2026). A recent meta-analysis of 1,337 studies reported comparisons of scores on eight empirically derived scales and sum scores of total, internalizing, and externalizing problems of young people with and without CPHCs (Pinquart, 2026). The meta-analysis revealed, on average, moderate elevations in internalizing problems (*g* = 0.51 standard deviation units) and small-to-moderate elevations in externalizing problems (*g* = 0.25) and total behavior problems (*g* = 0.49) compared to peers without CPHCs or test norms. The strongest elevations were found in somatic complaints (*g* = 0.70) and social problems (*g* = 0.65), and the weakest elevation was observed in delinquent behavior (*g* = 0.33).

No meta-analysis has been conducted on the results of the DSM-oriented scales in children and adolescents with CPHCs. Synthesizing these research findings is important for deciding whether to use the empirically derived and/or the DSM-oriented scales when working with children with CPHCs. Such a synthesis is also of practical relevance, as these scales provide more specific guidance for the focus of subsequent clinical structured interviews and intervention targets than the empirically based scales do (Bellina et al., 2013; de Wolff et al., 2014). Thus, the goal of the present study was to conduct the first meta-analysis of the results from DSM-oriented scales in children and adolescents with CPHCs. First, we asked whether children and adolescents with CPHCs would have higher scores on the six DSM-oriented scales than their peers without CPHCs or test norms.

Second, we were interested in the moderating effects of study characteristics. Since the empirically derived and DSM-oriented scales use items from the same pool, similar moderator effects may be found in both meta-analyses. However, the present study may find fewer significant moderator effects due to the smaller number of available studies, which leads to lower statistical power. For example, the DSM-oriented scales were introduced 18 years after the empirically derived scales.

The previous meta-analysis (Pinquart, 2026) found stronger elevations in internalizing problems (i.e., the anxious/depressed, withdrawn/depressed and somatic complaints scales) than in externalizing problems (i.e., delinquent and aggressive behavior). Elevations of symptoms also varied between individual CPHCs with the strongest externalizing problems occurring in cases of thalassemia and the strongest internalizing problems occurring in cases of chronic fatigue syndrome. Effect sizes were, in part, larger in cases of longer duration of the CPHCs and in studies from economically less developed countries. Results also varied by sociodemographic characteristics with stronger effect sizes in older samples and in samples with higher percentage of female children. Regarding methodology, young people with CPHCs exhibited weaker elevations (compared to their peers without such conditions and test norms) if self-reports were used rather than parent reports or reports from teachers/formal caregivers. In addition, effect sizes were, in part, larger if studies compared with healthy children rather than test norms, in studies with confirmed demographic equivalence of the compared groups, in convenience samples rather than probability samples, and if response rates were higher. In contrast, publication status, year of publication, and year of presentation did not moderate the results. We were interested in whether similar moderating effects would be found in studies with the DSM-oriented scales.

## 2 Research questions

In sum, the present meta-analysis addressed the following research questions: First, do children and adolescents with CPHCs exhibit higher scores on the DSM-oriented ASEBA scales (Affective Problems, Anxiety Problems, Somatic Problems, ADH Problems, Oppositional Defiant Problems, and Conduct Problems) compared to young people without CPHCs or when compared against test norms? Second, as the previous meta-analysis found stronger elevations of internalizing as compared to externalizing problems (Pinquart, 2026), we asked whether people with CPHC show greater elevations in the Affective Problems, Anxiety Problems, and Somatic Problems scales than in the other DSM-oriented scales? Third, are the effect sizes larger when observer reports (from parents or teachers) rather than (adolescent) self-reports are used, and do the results vary between CPHCs? Fourth, are the effect sizes stronger in case of longer CPHC duration, in older samples, in samples with a higher percentage of female participants, and in studies from less economically developed countries? As an anonymous reviewer suggested, we also tested whether stronger effect sizes would be found in CPHCs involving the nervous system directly and in visible CPHCs. Finally, are there moderating effects of publication status, year of publication, use of healthy peers versus test norms comparison, and study quality, such as the use of probability samples and demographically matched control groups?

## 3 Methods

### 3.1 Sample and coding

Consistent with the meta-analysis on the empirically derived ASEBA scales (Pinquart, 2026), we searched for studies through the electronic databases PsycInfo, MEDLINE, Google Scholar, and PSYNDEX. The terms” DSM-oriented” and “DSM-based” were added to the original search terms, leading to [(chronic health condition OR chronic illness OR disability OR AIDS OR arthritis OR asthma OR cancer OR chronic fatigue syndrome OR cleft OR cystic fibrosis OR deaf OR diabetes OR epilepsy OR headache OR heart disease OR hearing impairment OR HIV OR inflammatory bowel disease OR kidney disease OR liver disease OR migraine OR rheumatism OR sickle cell OR spina bifida OR visual impairment) and (Child Behavior Checklist OR CBCL OR Youth Self-Report OR Teacher Report Form OR Achenbach System of Empirically Based Assessment OR ASEBA) AND (DSM-based OR DSM-oriented)]. In addition, the reference sections of the identified studies were checked for additional papers. The final search was completed on June 1, 2026.

Inclusion criteria were (a) use of the DSM-oriented ASEBA scales, (b) comparison of DSM-oriented scores between children/adolescents with CPHCs and peers without these conditions or test norms, or provision of sufficient information for a comparison with established norms (e.g., by reporting the percentage above the clinical cut-off), (c) mean age of the sample of ≤ 18 years, and (d) provision of sufficient information for computing standardized between-group differences.

Exclusion criteria were (a) mixing data on CPHCs and other health conditions (such as curable acute infections), (b) selecting only young people with elevated emotional and behavioral problems, (c) duplicating results that had already been included, (d) reporting only effects of psychosocial interventions aimed at reducing emotional and behavioral problems, and (e) if we could not find a control group from the respective country for comparison. No language restrictions were applied, and we used help from native speakers and electronic translation software if we did not speak the respective language. To reduce the risk of bias due to nonpublication of nonsignificant results, we also included unpublished studies (e.g., dissertations) that were identified through the literature search. If no full text was available from national libraries, we tried to contact the authors by e-mail. The PRISMA flow chart of the search for studies is provided in Figure 1, the PRISMA Checklist in the electronic Supplementary material 1, and information on the included studies in Supplementary materials 2, 3.

**Figure 1:**
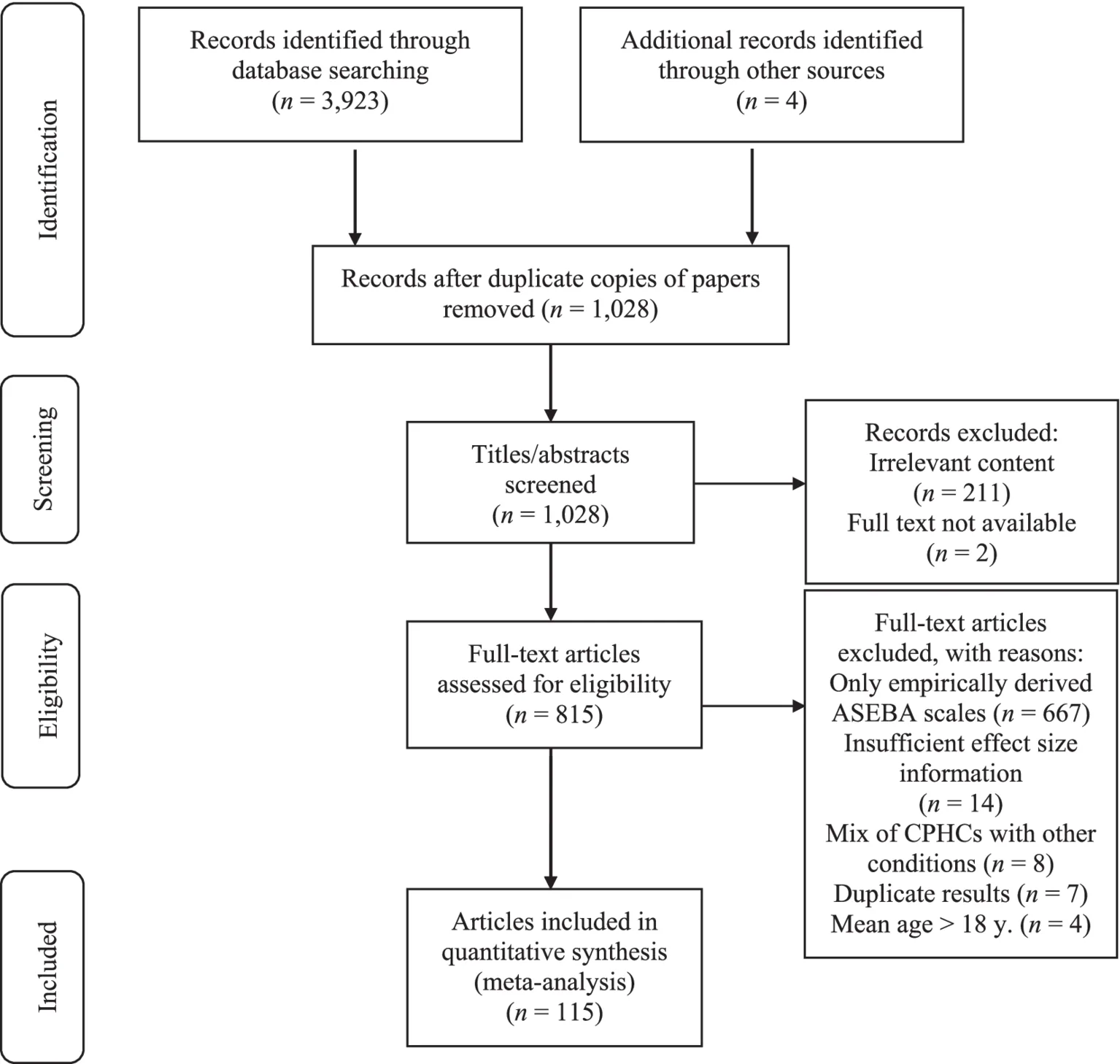
PRISMA 2009 flow diagram. Articles included in quantitative synthesis (meta-analysis).

We coded the following information from the individual studies: number of children/adolescents with CPHCs and members of the control condition, type of CPHC, the rater of emotional and behavioral problems (1 = parent, 2 = adolescent, 3 = teacher), direct neurological involvement (0 = no, 1 = yes), visibility of the CPHC (0 = low or only in some cases, 1 = always high), duration of the condition (in years), mean age, percentage of girls, percentage of members of ethnic minorities (based on the ethnic distribution in the country of the study), country of data collection, publication status (0 = unpublished, 1 = published), year of publication/presentation, sampling procedure (0 = convenience samples, 1 = probability samples), comparator (1 = children without CPHCs, 2 = test norms), study quality (see below), the percentage of children above the cut-off point for clinical problems, and the standardized size of between-group differences in Affective Problems, Anxiety Problems, Somatic Problems, ADH Problems, Oppositional Defiant Problems, and Conduct Problems.

For assessment of study quality and risk of bias, respectively, we used four criteria from the Mixed Methods Appraisal Tool for evaluating quantitative non-randomized studies (MMAT; Hong et al., 2018), namely whether participants were representative of the target population (as indicated by use of a probability sample and a response rate of ≥ 80%), whether valid outcome measures were used, whether complete effect size information were reported, and whether the risk of confounders was low (as indicated by sociodemographic equivalence of individuals with CPHC and the control condition). We could not apply the fifth criterion which exclusively refers to intervention studies. We chose the MMAT over longer instruments because we wanted to test the moderating effects of individual aspects of study quality and limit the risk of bias due to multiple testing. Information from the International Monetary Fund (International Monetary Fund, 2023) was used for categorizing countries as economically developed (countries with an advanced economy) or developing (emerging market and developing economy).

Both authors independently searched for relevant studies. Sixty studies were coded by both authors, and the remaining studies were coded by the first author. The number of double-coded studies far exceeds the minimum required for a robust assessment of inter-rater reliability (*n* = 21; Bonett, 2002). A mean intraclass correlation coefficient of 0.96 was established for continuous variables and there was 100% agreement for categorical variables. Discrepancies were resolved by double-checking the papers. The meta-analysis has been preregistered,^1^ and a review protocol is available from the first author.

### 3.2 Data analysis

Consistent with the previous meta-analysis, we computed standardized differences of the ASEBA scores of young people with CPHC and the control condition (Cohen’s *d*). Then, we adjusted the effect size estimates for small sample bias by computing Hedges’ *g.* For limiting the impact of outliers on the estimated mean effect size, effect sizes that differed by more than two standard deviations (*SDs*) from the mean were recoded to two *SDs* (Borenstein et al., 2021). As 21 studies provided more than one effect size per scale (e.g., in the case of longitudinal data, different raters or comparison of different CPHCs), we computed a multi-level meta-analysis that accounts for this dependence of data. This approach considers that individual effect sizes are nested within samples, which are nested within studies. It splits the total variance in between-study variance, variance between samples of the same study, and variance within samples. Thus, the within-subject variance (that is usually restricted in cases of multiple testing) is separated from between-subject and between-study variance.

We computed random-effects models that account for variation in the estimated effect sizes due to true differences as well as sampling error. Publication bias was addressed by multi-level Egger’s Regression test, and by directly comparing results from published and unpublished studies. We computed mean effect sizes, their confidence intervals, and tested for heterogeneity. Moderator analysis was performed with meta-regression. The analyses were computed with metafor in R (Viechtbauer, 2010). Effect sizes were interpreted as small (*g* = 0.20), moderate (*g* = 0.50), or large (*g* = 0.80), based on suggestions by Cohen (1992).

## 4 Results

### 4.1 Descriptive results

As shown in Figure 1, 115 studies met the inclusion criteria. These studies included 12,958 children and adolescents with CPHCs who had a mean age of 10.45 years (*SD* = 3.76). Approximately 45.5% were female and 26.9% belonged to an ethnic minority. In total, 24,638 children were members of the control group. The studies most often addressed epilepsy (18 studies), followed by atopic diseases (such as atopic dermatitis; 12 studies), cardiovascular diseases (11 studies), cancer (10 studies), HIV infection/AIDS (7 studies), obesity (6 studies), traumatic brain injury (6 studies), and chronic pain (5 studies). Some conditions were found using the broad search terms (e.g., chronic illness) without having specified them as search strings.

Parent reports using the CBCL (Achenbach and Rescorla, 2001) were collected in 105 studies: Twelve studies collected adolescent self-reports (with the Youth Self Report; Achenbach and Rescorla, 2001), and only four studies used the Teacher Report Form (Achenbach and Rescorla, 2001). In total, 88 studies were conducted in countries classified as developed by the International Monetary Fund (2023).

In terms of study quality and risk of bias, 100% of the studies had used a valid measure of the child’s psychological symptoms (i.e., the ASEBA) and 92.6% reported complete outcome data. However, only 26.5% confirmed the demographic equivalence of the compared groups based on child age and gender, and a response rate of 80% or higher, and only 14.8% used data from community-based random samples rather than convenience samples.

### 4.2 Mean between-group differences

Children and adolescents with CPHCs had higher scores on the six DSM-oriented problem scales than the control group. The largest between-group differences were observed in Somatic Problems (*g* = 0.658), Affective Problems (*g* = 0.612), and Anxiety Problems (*g* = 0.500; Table 1). These differences can be interpreted as moderate (Cohen, 1992). However, the between-group differences in ADH Problems (*g* = 0.429), Conduct Problems (*g* = 0.399), and Oppositional Defiant Problems (*g* = 0.380) did not reach the threshold for moderate effect sizes. As indicated by the non-overlap of the 95%-confidence intervals [CIs], young people with CPHCs showed significantly larger elevations in Somatic and Affective Problems than in ADH Problems, Conduct Problems, and Oppositional Defiant Problems. Between 14.33% (Affective Problems) and 8.63% (ADH Problems) of children with CPHCs scored in the clinical range (Table 1). These numbers are higher than the 2.3% of the general population who tend to score above the clinical cut-off (T-score ≥ 70; Achenbach, 2013). We found significant heterogeneity for each outcome.

**Table 1:** Weighted mean effect sizes, heterogeneity and percentages above the clinical cut-off for the six DSM-oriented ASEBA scales.

Conditions	*N*	*k*	*g*	*LL*	*UL*	*t*	*Q*	Level-1 variance	Level-2 variance	Level-3 variance	% Clinical
Affective Problems	181	137	0.612	0.532	0.692	15.16^***^	1,155.86^***^	0.0195^***^	0.0000	0.1215^***^	14.33
Anxiety Problems	168	132	0.500	0.425	0.577	13.03^***^	887.64^***^	0.0137^**^	0.0000	0.1033^***^	12.43
Somatic Problems	101	83	0.658	0.563	0.754	13.72^***^	394.05^***^	0.0065	0.0000	0.1071^***^	10.52
Attention Deficit/ Hyperactivity Problems	152	125	0.429	0.347	0.511	10.33^***^	740.66^***^	0.0141^***^	0.0000	0.1110^***^	8.63
Oppositional Defiant Problems	145	115	0.380	0.297	0.463	9.06^***^	631.65^***^	0.0000	0.0052	0.1129^***^	9.68
Conduct Problems	107	88	0.399	0.307	0.490	8.66^***^	459.77^***^	0.0000	0.0100	0.0968^***^	10.29

N, number of effect sizes; k, number of samples; g, weighted mean effect size; LL/UL, lower/upper level of the 95%-confidence interval; t, test for significance of g; Q, test for heterogeneity; % clinical, percent above the clinical cut-off, ^**^ *p* < 0.01, ^***^ *p* < 0.001.

Multi-level Egger’s test found evidence for a possible file-drawer problem for four scales (Affective Problems: *t*(181) = 3.12, *p* < 0.002; Anxiety Problems: *t*(170) = 3.53, *p* < 0.001; ADH Problems: *t*(151) = 3.73, *p* < 0.001; Oppositional-Defiant Problems: *t*(144) = 2.93, *p* < 0.004).

### 4.3 Moderator analysis

Meta-regression identified three rater effects. Between-group differences in Affective and Anxiety Problems were smaller when adolescent ratings were used rather than parent ratings (Table 2). In addition, between-group differences in Somatic Problems were smaller when teacher ratings were used instead of parent ratings.

**Table 2:** Moderator effects of sample and study characteristics.

Predictors	Affective problems				Anxiety problems				Somatic problems			
	estimate	*SE*	*df*	*t*	estimate	*SE*	*df*	*t*	estimate	*SE*	*df*	*t*
Rater	*F*(2,179) = 4.28, *p* < 0.05				*F*(2,167) = 9.76, *p* < 0.001				*F*(2,98) = 3.52, *p* < 0.04			
Adolescent^1^	−0.198	0.069	179	−2.88^**^	−0.245	0.056	167	−4.39^***^	−0.091	0.082	98	−1.10
Teacher^1^	−0.080	0.126	179	−0.64	−0.061	0.100	167	−0.60	−0.790	0.316	98	−2.50^*^
Kind of CPHC	*F*(8,173) = 0.96, *p* < 0.47				*F*(8,162) = 1.32, *p* < 0.24				*F*(8,92) = 1.14, *p* < 0.34			
Atopic conditions^2^	−0.176	0.126	173	1.40	−0.073	0.117	162	−0.63	0.023	0.165	92	0.14
Cancer^2^	0.104	0.151	173	0.69	0.067	0.148	162	0.45	0.107	0.198	92	0.54
Cardiovascular condit.^2^	−0.061	0.143	173	0.43	−0.140	0.134	162	−1.05	−0.177	0.168	92	−1.05
Chronic pain^2^	0.193	0.203	173	0.95	0.202	0.179	162	1.13	0.337	0.204	92	1.65
Epilepsy^2^	0.115	0.120	173	0.96	0.140	0.117	162	1.20	−0.147	0.132	92	−1.11
HIV infection^2^	0.003	0.155	173	0.02	−0.235	0.145	162	−1.62	0.141	0.171	92	−0.82
Obesity^2^	0.156	0.176	173	0.90	0.058	0.171	162	0.34	−0.036	0.244	92	−0.16
TBI^2^	0.166	0.192	173	0.87	0.177	0.160	162	1.10	0.224	0.234	92	0.96
Direct neurol. Involvement	0.243	0.084	180	2.90^**^	0.278	0.077	169	3.61^***^	−0.054	0.096	95	−0.56
High visibility	0.052	0.081	180	0.64	0.095	0.083	169	1.14	0.024	0.110	95	0.21
Duration of CPHC	−0.019	0.017	72	−1.14	−0.015	0.016	66	−0.94	−0.054	0.020	28	−2.69^*^
Mean age	0.023	0.009	177	2.39^*^	0.017	0.009	166	1.92	0.020	0.018	95	1.09
% female participants	0.001	0.002	162	0.25	−0.002	0.001	153	−1.45	0.002	0.002	90	1.03
Economically developed countries	−0.144	0.096	179	−1.51	−0.011	0.091	168	−0.12	0.026	0.103	98	0.25
Published	0.046	0.170	180	0.27	−0.025	0.167	169	−0.15	−0.034	0.267	99	−0.13
Year of publicat./presentation	−0.002	0.008	180	−0.21	0.007	0.008	169	0.89	−0.001	0.010	99	−0.07
Comparison with test norms	0.121	0.080	180	1.51	0.086	0.077	169	1.11	0.213	0.093	99	2.28^*^
Probability sample	−0.281	0.121	180	−2.34^*^	−0.180	0.114	169	−1.59	−0.235	0.180	99	−1.31
Matched samples	−0.052	0.085	180	−0.61	−0.013	0.082	169	−0.16	−0.214	0.092	98	−2.32^*^
Response rate ≥ 80%	0.056	0.081	180	0.69	0.073	0.077	169	0.96	0.005	0.103	99	0.05
Complete reporting	0.055	0.156	180	0.35	−0.106	0.152	169	−0.70	0.122	0.168	95	0.72

Predictors	Attention Deficit/Hyperact. Probl.				Oppositional Defiant Problems				Conduct Problems			
	estimate	*SE*	*df*	*t*	estimate	*SE*	*df*	*t*	estimate	*SE*	*df*	*t*
Rater	*F*(2,149) = 1.30, *p* < 0.28				*F*(2,142) = 0.08, *p* < 0.93				*F*(2,105) = 0.01, *p* = 0.99			
Adolescent^1^	−0.130	0.081	149	−1.60	−0.022	0.059	142	−0.36	0.004	0.072	105	0.06
Teacher^1^	0.011	0.148	149	0.07	0.016	0.125	142	0.13	−0.029	0.257	105	−0.11
CPHC	*F*(8,143) = 2.04, *p* < 0.05				*F*(8,136) = 1.85, *p* < 0.075				*F*(8,99) = 2.10, *p* < 0.05			
Atopic conditions^2^	−0.106	0.138	143	−0.80	−0.281	0.133	136	−2.12^*^	−0.164	0.135	99	−1.21
Cancer^2^	0.187	0.174	143	1.08	−0.044	0.194	136	−0.23	0.271	0.202	99	1.34
Cardiovascular cond.^2^	−0.160	0.140	143	−1.14	−0.228	0.135	136	−1.69	−0.142	0.149	99	−0.95
Chronic pain^2^	0.034	0.206	143	0.16	0.009	0.203	136	0.05	−0.132	0.192	99	−0.67
Epilepsy^2^	0.304	0.118	143	2.57^*^	0.094	0.118	136	0.80	0.175	0.117	99	1.50
HIV infection^2^	−0.187	0.145	143	−1.29	−0.272	0.144	136	−1.89	−0.117	0.162	99	−0.72
Obesity^2^	−0.008	0.171	143	−0.04	−0.022	0.160	136	−0.13	0.082	0.198	99	0.41
TBI^2^	0.203	0.180	143	1.13	0.390	0.272	136	1.43	0.567	0.254	99	2.23^*^
Direct neurol. Involvement	0.410	0.082	151	5.02^***^	0.035	0.086	143	3.76^***^	0.255	0.088	106	2.90^**^
High visibility	0.022	0.090	151	0.24	0.080	0.089	143	0.91	0.076	0.112	106	0.68
Duration of CPHC	−0.004	0.018	57	−0.24	−0.014	0.019	48	−0.72	−0.086	0.016	30	−5.33^***^
Mean age	0.030	0.009	146	3.24^**^	0.024	0.099	139	2.63^**^	−0.001	0.017	102	−0.07
% female participants	0.001	0.002	136	0.86	−0.001	0.001	128	−0.52	−0.000	0.002	89	−0.21
Economically developed countries	0.018	0.097	149	0.19	−0.018	0.097	142	−0.19	−0.048	0.103	105	−0.46
Published	−0.129	0.194	151	−0.67	−0.097	0.193	143	−0.50	−0.130	0.227	106	−0.57
Year of publicat./presentation	0.011	0.008	151	1.34	0.001	0.009	143	0.02	−0.003	0.010	106	−0.36
Comparison with test norms	0.429	0.042	151	10.33^***^	0.036	0.085	143	0.43	0.157	0.091	106	1.72
Probability sample	−0.187	0.124	151	−1.51	−0.150	0.129	143	−1.17	−0.194	0.147	106	−1.31
Matched samples	−0.027	0.088	151	−0.31	0.024	0.085	143	0.28	−0.005	0.091	105	−0.05
Response rate ≥ 80%	−0.020	0.080	151	−0.25	−0.032	0.068	143	−0.48	−0.002	0.094	105	−0.02
Complete reporting	−0.099	0.173	151	−0.57	−0.084	0.153	143	−0.55	−0.128	0.156	105	−0.82

SE, standard error; df, degrees of freedom; t, significance of the moderator. ^*^ *p <* 0.05, ^**^ *p* < 0.01, ^***^ *p* < 0.001.

1 Comparator: parents.

2 Comparator: other CPHCs.

We compared eight CPHCs for which at least five studies were available. The remaining studies served as a basis for comparison. Only three significant condition-specific statistical effects were found. Larger group differences in ADH problems were found in the case of epilepsy. Weaker group differences in Oppositional Defiant Problems were observed in young people with atopic conditions. Finally, larger group differences in Conduct Problems were detected in cases of traumatic brain injury (Table 2). The weighted mean effect sizes for the eight compared CPHCs are depicted in ESM5. Young people with atopic conditions, epilepsy, and obesity had significantly higher scores than the control condition on all six DSM-oriented scales. Children with cancer, cardiovascular conditions, and traumatic brain injury had significantly higher scores on four out of six scales, while children with chronic pain and HIV-infection/AIDS showed higher scores on three scales.

In CPHCs with direct neurological involvement, we found higher levels of psychological problems on the scales except Somatic Problems than in other CPHCs. In contrast, the visibility of the CPHCs had no moderating effect.

We found two moderating effects of CPHC duration. The effect sizes of Somatic Problems and Conduct Problems were smaller if the CPHCs lasted longer. There were also three moderating effects of child age. Studies on Affective Problems, ADH Problems, and Oppositional Defiant Problems found larger effect sizes in older samples. Since age and the duration of the CPHC were positively correlated (*r* = 0.31, *p* < 0.02), we tested whether the moderating effects of age and duration persist when both variables are included simultaneously. The moderating effect of age remained significant when statistically controlling for duration. Additionally, a longer CPHC duration was associated with smaller effect sizes on all DSM-oriented scales (estimates ranged from −0.04 to −0.083, *t*-values ranged from −2.08 to −5.20, *p* < 0.05), when statistically controlling for age differences.

The effect sizes of Somatic Problems and ADH Problems were larger when test norms were used for comparison rather than healthy children. We did not identify any moderating effects related to child gender, publication status, and year of publication/presentation. The results for economically developed countries and other countries were also similar.

Regarding study quality criteria, studies with probability samples found smaller effect sizes than those with (clinical) convenience samples for Affective Problems. Effect sizes for Somatic Problems were smaller when the compared groups were demographically matched. Finally, the response rate and complete reports of outcome data had no moderating effects.

## 5 Discussion

The present study provides the first meta-analysis of results from the six DSM-oriented ASEBA scales in young people with CPHCs. Regarding the first research question, we found that children and adolescents with CPHCs exhibit higher scores on the DSM-oriented ASEBA scales compared to peers without CPHC or against test norms. As addressed in the second research question, young people with CPHCs showed significantly larger elevations in Somatic and Affective Problems than in ADH Problems, Conduct Problems, and Oppositional Defiant Problems. Regarding the third research question, we observed, in part, stronger elevations of observer-rated problems compared to self-reported problems. Comparisons yielded largely similar results across the various CPHCs. Concerning sample characteristics (research question 4), there was evidence for stronger elevations of ASEBA scores in the case of CPHCs with neurological involvement, shorter duration of the CPHCs, and in older samples. In contrast, visibility of the CPHCs, child gender and the economic wealth of the country did not play a role. Finally, relating methods of the studies, we found, in part, stronger elevations if test norms rather than healthy comparison groups and convenience samples rather than probability samples were used and if the demographic equivalence of the compared groups was not confirmed. We begin our discussion by comparing results on the DSM-oriented ASEBA scales and the empirically derived scales from a previous meta-analysis (Pinquart, 2026).

Although the present meta-analysis included more than 100 studies, this number is more than 10 times smaller than the number of studies on the empirically derived scales (Pinquart, 2026). This difference cannot be explained solely by the later introduction of the DSM-oriented scales (in 2001 as compared to 1983; Achenbach and Edelbrock, 1983; Achenbach and Rescorla, 2001) as approximately 770 studies have used the empirically derived scales with young people experiencing CPHCs between 2011 and 2025 (Pinquart, 2026). Other reasons may include the availability of broad sum scales (i.e., internalizing, externalizing, total problems) when using the empirically derived scales, whereas such broad sum scales have not been defined for the DSM-oriented scales. Another reason may be the interest in empirically derived scales that have no equivalent in DSM-oriented scales, such as social problems.

Similar to the previous meta-analysis, the weighted between-group differences were small to moderate or moderate, respectively, when using Cohen’s (1992) interpretation guidelines. This similarity is not surprising, as the empirically derived and DSM-oriented scales use items from the same item pool. Although scoring in the clinical range on the ASEBA does not mean that a clinical diagnosis would be made based on a structured clinical interview (e.g., Chromik and Friedman, 2026), the reported numbers indicate that only a minority of young people with CPHCs—between 8.6% (ADH Problems) and 14.3% (Affective Problems)—may fulfill the criteria of a related disorder.

Multi-level Egger’s test suggests a possible file-drawer problem for four scales, although direct comparisons of the results from published and unpublished studies did not find significant differences. However, we cannot rule out the possibility that the results from unpublished studies identified in our research might differ from those of unpublished studies not found in the electronic databases.

When comparing the six DSM-oriented scales, we found larger effect sizes for Somatic and Affective Problems than for ADH Problems, Conduct Problems, and Oppositional Defiant Problems. The largest effect size for Somatic Problems can be explained by an overlap with symptoms of the CPHCs or treatment side effects (e.g., aches, nausea; Achenbach, 2013). The above-average effect size for Affective Problems may indicate a psychological response to losses associated with CPHCs, such as loss of control over one’s health or loss of positive activities, although symptom overlap may also play a role (e.g., when CPHC symptoms include tiredness and sleep problems). Although we analyzed only concurrent associations of CPHCs with ASEBA scores, longitudinal studies also showed that CPHCs predict emotional and behavioral symptoms at follow-up (Määttä et al., 2022). Elevations of ADH Problems, Oppositional Defiant Problems, and Conduct Problems can be partially explained by impairments in executive function that play a role only in a limited number of CPHCs, such as epilepsy (van den Berg et al., 2021).

Due to the smaller numbers of available studies that lead to lower test power, it is not surprising that we found fewer moderating effects than in the previous meta-analysis (Pinquart, 2026). Nonetheless, we again found some evidence of larger effect sizes when parent ratings of child problems were used rather than ratings from other sources. These differences were restricted to internalizing problems (Affective Problems, Anxiety Problems) that are more difficult to observe than externalizing problems (Hyperactivity, Oppositional Defiant and Conduct Problems) and are, therefore, more prone to subjective interpretations. In families with pediatric CPHC, parental reports on their own psychological distress tend to be higher than child reports on their own distress (Pinquart, 2019), and distressed parents may overestimate symptoms in their child. Alternatively, children may under-report their psychological distress due to unwillingness to acknowledge some social–emotional challenges or limited awareness (Brown et al., 2023). Although the meta-analysis of empirically derived scales revealed the highest levels of emotional and behavioral issues among young individuals with thalassemia and chronic fatigue syndrome (Pinquart, 2026), we could not identify any studies on these conditions that employed DSM-oriented scales. This may explain why we found very few condition-specific statistical effects. The higher ADH Problems observed in young people with epilepsy and the higher conduct problems observed in young people with traumatic brain injury may result from impaired executive function (van den Berg et al., 2021). However, self-control problems are also risk factors for traumatic brain injury (Brandt et al., 2024). The relatively lower Oppositional Defiant Problems in young people with atopic conditions, compared to other CPHCs, might indicate that some young people with atopic conditions avoid oppositional defiant behavior because the associated stress exacerbates inflammatory and autoimmune conditions – such as acne, psoriasis, and atopic dermatitis (Zhang et al., 2024).

When interpreting the result that, unlike cancer, chronic pain, and HIV infection, only young people with atopic conditions, epilepsy, and obesity showed elevated scores across all of the six DSM-oriented scales, we must be aware that fewer studies were conducted on the former conditions, thus reducing statistical power. More significant effect sizes may be found when more studies on these conditions are available.

While the higher levels of ADH Problems, Oppositional Defiant Problems, and Conduct Problems in CPHCs with direct neurological involvement can be explained by associated deficits in executive function (van den Berg et al., 2021), other factors could also play a role in the elevated Affective Problems and Anxiety Problems, such as loss of control over body functions or social stigma (Whelan and Walker, 2009). The lack of moderating effects of visibility may result from problems with coding this variable at the study level (e.g., when including individuals with and without active epileptic seizures). Alternatively, visibility may be too non-specific, as for example, negative social consequences are particularly found in those visible CPHCs that strongly impair perceived attractiveness (Pinquart, 2017).

We found, in part, larger effect sizes when CPHCs lasted for shorter and participants were older. As the previous meta-analysis suggested, some CPHC-related stressors may emerge at a later age. For instance, CPHCs may impede the development of adolescent autonomy or their building of romantic relationships (Jones et al., 2022). Information on the duration of CPHCs was most often available for congenital conditions (e.g., cleft lip and palate and congenital heart defects that underwent surgical correction in early childhood) and for cancer. In most of these cases, a longer duration means a longer distance from the most stressful illness symptoms and treatments, which may explain the lower symptom levels associated with a longer interval since the onset of the CPHCs.

The two identified moderating effects of the comparator may indicate that older test norms underestimate the prevalence of ADH Problems and Somatic Problems in the recent general population, possibly because of the Covid-19 pandemic or other recent stressors (Conti et al., 2020; Rogers and MacLean, 2023).

As only one out of six analyses found a moderator effect of the use of probability versus convenience samples and of demographic equivalence of the compared groups, these moderating effects should be interpreted with caution. Nonetheless, community-based probability samples may be more likely to include less severe cases than convenience samples of treatment-seeking young people. This could lead to somewhat lower effect sizes in the former studies. The moderating effect of using matched samples indicates that comparisons with other groups are more error prone as the compared groups may differ in third variables.

The lack of moderating effects of the country’s economic situation contradicts previous findings (Pinquart, 2026). Since only 23.7% of the studies included in the present meta-analysis were conducted in economically less developed countries (International Monetary Fund, 2023) and since the test power of the present study was lower than that of the previous meta-analysis, we may not have been able to detect small national differences. Finally, the absence of moderating effects of study quality characteristics suggests that such effects, if existent, were too small to be detected.

## 6 Limitations and conclusions

There are some limitations to the present meta-analysis that must be mentioned. First, the number of included studies was much lower than in a recent meta-analysis of empirically derived ASEBA scores (Pinquart, 2026). Specifically, two conditions that were found to be associated with the highest CBCL sum-scale scores (thalassemia and chronic fatigue syndrome) were not represented in the present meta-analysis. Condition-specific analyses could only be computed for eight CPHCs. Second, the analysis of the concurrent associations of CPHCs with psychological problems does not permit causal interpretations. Third, most of the included data were based exclusively on parental reports (i.e., the CBCL). Fourth, we did not correct for multiple testing. Finally, we limited the moderator analysis to variables that had been consistently measured across a larger number of studies. Therefore, for example, we could not test the moderating effects of CPHC severity.

Despite these limitations, the following conclusions can be drawn. First, due to the discrepancy between the number of studies on the empirically derived and the DSM-oriented scales, more analyses of the DSM-oriented scales in children with CPHCs are recommended. Many data sets are already available for such an analysis. Second, results on Somatic Problems should be interpreted with caution due to the overlap with CPHC symptoms. Third, given that the effect sizes are sometimes larger when using parental reports of the child’s problems, the use of multiple raters provides a more complete picture. Fourth, we conclude that only a small minority of children with CPHCs are likely to meet the criteria of psychiatric disorders related to the DSM-oriented ASEBA scales.

Fifth, the DSM-oriented scales are recommended as part of stepwise assessments that identify symptom domains to be targeted with a structured clinical interview, due to their greater specificity compared to the empirically derived scales (e.g., Bellina et al., 2013). It is not yet known whether the DSM-oriented or empirically derived scales would better differentiate between levels of psychological problems in children with and without CPHCs. Larger numbers of studies using both groups of scales on the same data sets are needed to answer this question.

Sixth, young people with conditions with direct neurological involvement are mostly in need of screening for psychological problems. Finally, a multidisciplinary team approach involving medical and psychosocial specialists could be implemented for screening elevated psychological symptoms and, if needed, conducting clinical interviews. This is because medical specialists are usually the first point of contact for children with CPHCs. These teams can treat physical and mental health conditions and prevent the negative psychological effects of CPHCs by promoting coping skills and other psychological resources (King et al., 2024).

## Data Availability

The original contributions presented in the study are included in the article/Supplementary material, further inquiries can be directed to the corresponding author.

## Footnotes

1
https://osf.io/nfvdj
