# Supplementary material for: Assessment of psychological problems in children with chronic physical health conditions using DSM-oriented ASEBA scales: a meta-analysis
Source: Front Psychol. 2026 Oct 16;17:1930737. doi: 10.3389/fpsyg.2026.1930737 (PMC13719451; doi:10.3389/fpsyg.2026.1930737)
Supplement: Supplementary file 1 [file Supplementary_file_1.docx]

**ESM 1: Selected Characteristics of the Studies Included**

| Authors | CPHC | N_CPHC_ | Mean age | % female | Scales |
| --- | --- | --- | --- | --- | --- |
| Abdelmonem et al. (2022) | hearing impairment | 25 | 3.50 | 24.0 | AF, AX, AD, O |
| Aboeisa et al. (2022) | epilepsy | 60 | 9.42 | 51.7 | AF, AX, S, AD, O, C |
| Ahmed et al. (2021) | epilepsy | 20 | 8.20 | 10.0 | AF, AX, S, AD |
| Al Ghriwati (2015) | asthma | 200 | 7.86 | 36.7 | AF, AX |
| Al-Khotani et al. (2018) | chronic pain/ temporomandibular disorders | 85 | 14.00 | 70.6 | AF, AX, S, AD, O, C |
| Ali et al. (2025) | epilepsy | 100 | 13.86 | 44.0 | AD, O, C |
| Baggio et al. (2022) | childhood absences epilepsy | 10 | 10.16 | 43.8 | AF, AX, S, AD, O, C |
| Baggio et al. (2022) | epilepsy with centrotemporal spike | 12 | 10.16 | 43.8 | AF, AX, S, AD, O, C |
| Baggio et al. (2022) | Panayiotopoilos syndrome | 6 | 10.16 | 43.8 | AF, AX, S, AD, O, C |
| Bakri et al. (2014) | sickle cell anemia | 35 | 3.51 | 48.6 | AF, AX, AD, O |
| Balottin et al. (2015) | chronic headache | 42 | 14.17 | 52.4 | AF, AX, S, AD, O, C |
| Battistutta et al. (2010) | tension-type headache | 48 | 13.40 | 66.7 | AF, AX, S, AD, O, C |
| Bemis et al. (2015) | cancer | 318 | 10.60 | 47.2 | AF |
| Bender et al. (2008) | epilepsy | 60 | 11.00 | 41.7 | AF, AX, S, AD, O, C |
| Berg et al. (2008) | epilepsy | 128 | 13.08 | 48.0 | AF, AX, S, AD, O, C |
| Bisacchi et al. (2011, 6-10-year-olds) | congenital hypothyroidism | 44 | 8.00 | . | AF, AX, S, AD, O, C |
| Bisacchi et al. (2011, 11-13-year-olds) | congenital hypothyroidism | 23 | 12.00 | . | AF, AX, S, AD, O, C |
| Bisacchi et al. (2011, 14-18-year-olds) | congenital hypothyroidism | 19 | 16.00 | . | AF, AX, S, AD, O, C |
| Boscolo (2017, HIV infection) | HIV infection | 23 | 14.00 | 52.2 | AF, AX, S, AD, O, C |
| Boscolo (2017, HSCT survivor) | mixed conditions (HSCT) | 31 | 16.00 | 38.7 | AF, AX, S, AD, O, C |
| Brandsma et al. (2019) | twin-to-twin-transfusion-syndrome | 417 | 2.00 | 49.4 | AF, AX, AD, O |
| Brumbaugh et al. (2023, intervention) | retinopathy | 71 | 2.00 | 40.8 | AF, AX, AD, O |
| Brumbaugh et al. (2023, no intervention) | retinopathy | 262 | 2.00 | 53.4 | AF, AX, AD, O |
| Chang et al. (2013) | allergic rhinitis | 141 | 4.80 | 48.7 | AF, AX, AD, O |
| Chang et al. (2013) | asthma | 40 | 4.80 | 48.7 | AF, AX, AD, O |
| Chang et al. (2013) | atopic dermatitis | 84 | 4.80 | 48.7 | AF, AX, AD, O |
| Chiou et al. (2019) | acute lymphoblastic leukemia | 18 | . | 38.1 | AD |
| Chira et al. (2018, early repair) | tetralogy of fallot | 21 | 6.88 | . | AF, AX, S, AD, O, C |
| Chira et al. (2018, later repair) | tetralogy of fallot | 37 | 6.88 | . | AF, AX, S, AD, O, C |
| Chisholm et al. (2022) | neurofibromatosis type 1 | 68 | 9.00 | 44.1 | AF, AX, O |
| Choi et al. (2016) | allergic rhinitis | 35 | 8.90 | 42.9 | AF, AX, S, AD, O, C |
| Choi et al. (2016) | asthma + allergic rhinitis | 29 | 9.40 | 37.9 | AF, AX, S, AD, O, C |
| Choi et al. (2016) | asthma | 27 | 10.40 | 40.7 | AF, AX, S, AD, O, C |
| Compass et al. (2014) | cancer | 385 | 10.60 | 52.0 | AF, AX |
| Crerand et al. (2024) | cleft lip & palate | 143 | 9.40 | 38.9 | AF, AX, AD |
| Crerand et al. (2024) | cleft lip | 46 | 9.40 | 39.1 | AF, AX, AD |
| Crerand et al. (2024) | cleft palate | 63 | 9.23 | 69.0 | AF, AX, AD |
| de Jong et al. (2020) | mixed conditions | 13 | 9.67 | 30.8 | AF, AX, S, AD, O, C |
| Dollman et al. (2017) | severe traumatic brain injury | 26 | 12.58 | 34.0 | AF, AX, S, AD, O, C |
| Duchoslav (2012) | acute lymphoblastic leukemia | 5 | 8.00 | 100 | AF, AX |
| Dufton et al. (2009) | recurrent abdominal pain | 21 | 11.05 | 57.1 | AF, AX, S |
| Eiffener et al. (2018) | obesity | 77 | 5.00 | 53.0 | AF, AX, AD, O |
| Familiar et al. (2015) | HIV infection | 144 | 8.90 | 50.0 | AF, AX, S, AD, O, C |
| Ferro et al. (2016a) | food allergy | 268 | 13.90 | 44.8 | AF, AX, S, AD, O, C |
| Ferro et al. (2016a) | food allergy | 268 | 13.90 | 44.8 | AF, AX, AD, O, C |
| Ferro et al. (2016b) | asthma | 1258 | 13.90 | . | AF, AX |
| Firoozi (2017) | hemophilia | 65 | 7.50 | . | AF, AX, S, AD, O |
| Fredericks et al. (2007) | liver disease | 38 | 8.50 | 51.6 | AF, AX, S, AD, O, C |
| Gaynor et al. (2009) | congenital heart disease | 380 | 4.50 | 44.0 | AF, AX, AD, O |
| Geerdink et al. (2020) | congenital heart disease | 35 | 13.00 | 53.0 | AF, AX, S, AD, O, C |
| Getsuwan et al. (2022, older group) | liver disease | 45 | 12.00 | 60.0 | AF, AX, S, AD, O, C |
| Getsuwan et al. (2022, younger group) | liver disease | 32 | 3.50 | 60.0 | AF, AX, S, AD, O, C |
| Goodwin et al. (2013) | asthma | 390 | 10.80 | 40.5 | AF, AX, S, AD, O, C |
| Goodwin et al. (2017, test only) | atopic diseases | 294 | 11.00 | . | AF, AX, S, AD, O, C |
| Goodwin et al. (2017, parental + test report) | atopic diseases | 268 | 11.00 | . | AF, AX, S, AD, O, C |
| Goodwin et al. (2017, parental report only) | atopic diseases | 185 | 11.00 | . | AF, AX, S, AD, O, C |
| Hecker (2016) | HIV infection | 54 | 8.26 | 53.7 | AF, AX, S, AD, O, C |
| Hernandez-Reif et al. (2007, massage group) | HIV infection | 24 | 5.08 | . | AF, AX, AD, O |
| Hernandez-Reif et al. (2007, play group) | HIV infection | 24 | 5.08 | . | AF, AX, AD, O |
| Hesdorffer et al. (2012, complicated) | epilepsy | 95 | 13.50 | . | AF, AX, AD, O, C |
| Hesdorffer et al. (2012, uncomplicated | epilepsy | 213 | 13.50 | . | AF, AX, AD, O, C |
| Hostert et al. (2015) | cancer | 18 | 9.40 | 22.4 | AF, AX, S, AD, C |
| Jantarabenjakul (2018) | HIV infection | 50 | 2.42 | 44.0 | AF, AX, AD, O |
| Johns et al. (2021) | craniofacial microsomia | 89 | 3.18 | 37.1 | AF, AX, AD, O |
| Kapp-Simon et al. (2012) | single-suture craniosynostosis | 219 | 1.56 | 35.0 | AF, AX, AD, O |
| Karver et al. (2012, mild/moderate) | traumatic brain injury | 63 | 7.94 | . | AX, AD |
| Karver et al. (2012, severe) | traumatic brain injury | 23 | 8.07 | . | AX, AD |
| Keenan et al. (2018, mild) | traumatic brain injury | 144 | 9.35 | 39.0 | AF, AX, AD |
| Keenan et al. (2018, mild/moderate) | traumatic brain injury | 156 | 9.48 | 37.8 | AF, AX, AD |
| Keenan et al. (2018, severe) | traumatic brain injury | 86 | 9.23 | 28.0 | AF, AX, AD |
| Kelmanson (2018) | cerebral palsy | 20 | 5.00 | 50.0 | AF, AX, AD, O, C |
| Lande et al. (2009) | primary hypertension | 32 | 14.00 | . | AF, AX, S, AD, O, C |
| Lepage et al. (2025) | congenital heart disease | 66 | 2.00 | 36.4 | AF, AX, AD, O, C |
| Lommel et al. (2009) | fibromyalgia | 32 | 15.20 | 100 | AF, AX, S, AD, O, C |
| Loutfi et al. (2011) | epilepsy | 30 | 9.08 | 50.0 | AF, AX, S, AD, O, C |
| Louw at al. (2016) | HIV infection | 78 | 10.26 | 52.6 | AF, AX, S, AD, O, C |
| Malucelli et al. (2024) | narcolepsy type 1 | 37 | 12.20 | 43.2 | AF, AX, S, AD, O, C |
| Mandell et al. (2022) | obesity | 32 | 14.19 | 68.6 | O |
| Mavrea et al. (2026) | cancer survivor | 62 | 11.72 | 52.5 | AF, AX, S, AD, O, C |
| Maxwell et al. (2017) | arterial ischemic stroke | 50 | 12.10 | . | AF, AX, S, O, C |
| Meldrum et al. (2012) | IgE mediated food allergy | 38 | 1.50 | . | AF, AX, S, AD, O, C |
| Meldrum et al. (2012) | Non-IgE mediated food allergy | 29 | 1.50 | . | AF, AX, AD, O |
| Mendizabal (2016, active) | benign childhood epilepsy | 10 | 11.30 | 16.7 | AF, AD, O, C |
| Mendizabal et al. (2025, in remission) | benign childhood epilepsy | 9 | 15.50 | 70.0 | AF, AD, O, C |
| Miller et al. (2009) | cancer | 75 | 10.08 | 43.0 | AF, AX |
| Moraes et al. (2023) | mild atopic dermatitis | 57 | 10.60 | 50.9 | AF, AX, S, AD, O, C |
| Moraes et al. (2023) | moderate to severe atopic dermatitis | 43 | 11.80 | 34.5 | AF, AX, S, AD, O, C |
| Morava et al. (2010) | other metabolic disorders | 18 | . | . | AF |
| Morava et al. (2010) | OXPHOS disease | 18 | 8.64 | 11.1 | AF |
| Moreira et al. (2014) | epilepsy | 45 | 11.00 | . | AF, AX, S, AD, O, C |
| Nagata et al. (2015) | obesity | 47 | 5.00 | . | AF, AX, AD, O |
| Nardi et al. (2008) | diabetes type 1 | 70 | 13.80 | 44.3 | AF, S, O, C |
| Nazari at al. (2014) | leukemia | 100 | 8.97 | 36.0 | AF, AF, AD, O |
| Neto et al. (2018) | phenylketonuria | 36 | 11.30 | 38.9 | AF, AX, S, AD, O, C |
| Nitra et al. (2020) | migraine | 18 | 12.00 | 31.2 | AX |
| Nunes et al. (2017) | sickle cell disease | 15 | 9.00 | 49.0 | AF, AX, S, AD, O, C |
| Operto et al. (2021) | epilepsy | 103 | 12.54 | 42.0 | AF |
| Palmieri et al. (2024) | arthritis | 30 | 9.77 | 56.7 | AF, AX, AD, O |
| Park, M. et al. (2016) | hearing impairment | 32 | . | . | AF, AX, S, AD, O, C |
| Park, S.H. et al. (2023, mild-to-moderate) | atopic dermatitis | 35 | 10.80 | 51.4 | AF, AX, S, AD, O, C |
| Park, S.H. et al. (2023, severe) | atopic dermatitis | 34 | 10.80 | 61.8 | AF, AX, S, AD, O, C |
| Parmeggiani et al. (2018) | neurofibromatosis type 1 | 36 | 9.00 | 47.2 | AF, AX, S, AD, O, C |
| Perez et al. (2018) | pseudohypoparathyroidismtype1A | 15 | 12.60 | 58.0 | AF, AX, S, AD, O, C |
| Peterson et al. (2013, moderate) | traumatic brain injury | 79 | 14.49 | 32.9 | AF, AX, S |
| Peterson et al. (2013, severe) | traumatic brain injury | 51 | 14.91 | 37.3 | AF, AX, S |
| Pound et al. (2012, younger than 5 years) | leukemia | 16 | 3.00 | 51.2 | AF, AX, S, AD, O, C |
| Pound et al. (2012, 5+ years) | leukemia | 27 | 9.80 | 51.2 | AF, AX, S, AD, O, C |
| Pride et al. (2023) | neurofibromatosis type 1 | 152 | 8.10 | 46.0 | AF, AX |
| Prussien et al. (2018) | sickle cell disease | 44 | 9.30 | 43.2 | AF |
| Rackauskaite et al. (2016) | cerebral palsy | 446 | 12.25 | 43.0 | AF, AXAD, O |
| Rocca et al. (2016) | epilepsy | 39 | 10.64 | 49.0 | AF, AX, S, AD, O, C |
| Rocca et al. (2016) | narcolepsy type 1 | 29 | 11.52 | 45.0 | AF, AX, S, AD, O, C |
| Roessler et al. (2021) | Cantu syndrome | 18 | 8.50 | . | AF, AX, AD |
| Rofey et al. (2015, control group) | obesity | 82 | 16.11 | 81.9 | AF, AX, S, AD, O, C |
| Rofey et al. (2015, intervention group) | obesity | 137 | 16.86 | 79.9 | AF, AX, S, AD, O, C |
| Sady et al. (2025) | celiac disease | 34 | 13.01 | 56.0 | AD |
| Saini (2023) | cardiovascular conditions | 7 | 4.50 | 42.9 | AF, AX, AD, O |
| Sarrechia et al. (2015) | atrial septal defect | 48 | 9.25 | 60.4 | AF, AX, S, AD, O, C |
| Shinnar et al. (2017) | epilepsy | 382 | 7.60 | 57.0 | AF, AX, S, AD, O, C |
| Singh et al. (2012) | asthma | 50 | 9.62 | . | AF, AX, S, C |
| Singh et al. (2012) | epilepsy | 100 | 9.64 | . | AF, AX, S, C |
| Skokauskas et al. (2012) | Prader-Willi Syndrome (PWS) | 24 | 9.92 | 45.8 | AF, AX, S, AD, O, C |
| Soedjana et al. (2022) | cleft lip & palate | 104 | 8.00 | 43.3 | AF, AX, S, AD, O, C |
| Stavropoulos et al. (2017) | epilepsy | 22 | .00 | 40.9 | AF, AX, S, AD, O, C |
| Taddei et al. (2019) | neurofibromatosis type 1 | 25 | 9.33 | 46.2 | AD, O |
| Taddei et al. (2019) | NF-1 & optical pathways glioma | 24 | 7.54 | 57.7 | AD, O |
| Taddei et al. (2019) | NF-1 & other cancer | 16 | 8.66 | 46.5 | AD, O |
| Tester et al. (2021) | supraventricular tachycardia | 29 | 12.63 | 60.0 | AF, AX, S, AD, O, C |
| Tremolada et al. (2016) | diabetes type 1 | 45 | 13.19 | 55.9 | C |
| Uhlig (2023) | Robin sequency | 21 | 9.7 | 48.0 | AF, AX, S, AD, O, C |
| Usubini et al. (2024) | obesity | 100 | 15.30 | 64.0 | AF, AX, S, AD, O, C |
| Usubini et al. (2025) | idiopathic growth hormone deficit | 10 | 10.90 | 60.0 | AF, AX, S, AD, O, C |
| van der Mheen et al. (2020, older group) | dilated cardiomyopathy | 31 | 12.40 | 45.2 | AF, AX, S, AD, O, C |
| van der Mheen et al. (2020, younger group) | dilated cardiomyopathy | 37 | 2.20 | 45.9 | AF, AX, AD, O |
| van der Plas et al. (2017) | leukemia | 23 | 14.40 | 0 | AF, AX, S, AD, O, C |
| Van Vliet et al. (2019, 8-12-year-olds) | tyrosinemia type 1 | 15 | 9.29 | 38.7 | AF, AX, S, AD, O, C |
| Van Vliet et al. (2019, 13-17-year-olds) | tyrosinemia type 1 | 9 | 15.62 | 33.3 | AF, AX, S, AD, O, C |
| Vaz et al. (2026, mild) | asthma | 33 | 11.00 | 45.5 | AF, AX, S, AD, O, C |
| Vaz et al. (2026, moderate-severe) | asthma | 67 | 10.00 | 49.3 | AF, AX, S, AD, O, C |
| Visser et al. (2018) | HIV infection | 54 | 8.26 | 53.7 | AF, AX, S, AD, O, C |
| Vourdoumpa et al. (2026) | obesity | 236 | 10.26 | . | AF, AX, S, AD, O, C |
| Wade et al. (2020, mild-moderate, female) | traumatic brain injury | 127 | 12.20 | 100 | AF, AX, S, AD, O, C |
| Wade et al. (2020, mild-moderate, male) | traumatic brain injury | 222 | 12.20 | 0 | AF, AX, S, AD, O, C |
| Wade et al. (2020, severe, female) | traumatic brain injury | 76 | 12.20 | 100 | AF, AX, S, AD, O, C |
| Wade et al. (2020, severe, male) | traumatic brain injury | 147 | 12.20 | 0 | AF, AX, S, AD, O, C |
| Wahab et al. (2019) | celiac disease | 51 | 5.90 | 64.7 | AF, AX, AD, O |
| Wolock et al. (2020) | mixed diseases | 71 | 10.79 | 48.5 | AF, AX |
| Yahia et al. (2026) | neurofibromatosis type 1 | 29 | 9.20 | 47.4 | AF, AX, S, AD, O, C |
| Yamul et al. (2024) | traumatic brain injury | 13 | 3.60 | 69.2 | AF, AX, AD, O |
| Zambrelli et al. (2020) | epilepsy | 84 | 12.00 | 46.4 | AF, AX, S, AD, O, C |
| Zambrelli et al. (2021) | tuberous sclerosis complex | 46 | 9.96 | 56.5 | AF, AX, S, AD, O, C |
| Zanaboni et al. (2026) | epilepsy | 68 | 14.50 | 65.0 | AF, AX, S, AD, O, C |
| Zanato et al. (2017) | mixed (leukemia, lymphoma, others) | 36 | 14.40 | 37.8 | AF, AX, S, AD, O, C |
| Zeller et al. (2006) | extreme obesity | 33 | 16.30 | . | AF |
| Zoair et al. (2015) | heart diseases | 23 | 9.40 | . | AF, AX, S, AD, O, C |
| Zoair et al. (2015) | rheumatic heart disease | 24 | 10.80 | 58.3 | AF, AX, S, AD, O, C |

*Note*. AF = Affective Problems, AX = Anxiety Problems, S = Somatic Problems, AD = Attention Deficit Hyperactivity Problems, O = Oppositional Defiant Problems, C = Conduct Problems.

**ESM 2: References of the Included Studies**

Abdelmonem, A. A., Salah, H., Mostafa, H. A., Abd ElMonem, N. A., Khalil, D. M., Youssef, R. S., & Fahiem, R. A. (2022). Assessment of behavioral problems in children pre-and post-cochlear implant: An Egyptian study. *Psychiatry Investigation*, *19*(9), 763. https://doi.org/10.30773/pi.2022.0041

Aboeisa, M. I., Seleem, M. A. H., El Gohary, T. M., & Al Shahawy, A. K. (2022). Study of cognitive and behavioral impacts of idiopathic epilepsy and antiepileptic drugs (AEDs) in children and adolescents. *Journal of Advances in Medicine and Medical Research*, *34*(19), 176-187. https://doi.org/10.9734/JAMMR/2022/v34i1931451

Ahmed, G. K., Darwish, A. M., Khalifa, H., & Khashbah, M. A. (2021). Evaluation of psychiatric comorbidity in attention-deficit hyperactivity disorder with epilepsy: A case-control study. *Epilepsy Research*, *169*, 106505. https://doi.org/10.1016/j.eplep syres.2020.106505

Al Ghriwati, N. (2015). *Using structural equation modeling to understand the role of the family in pediatric asthma contexts* (Unpublished master thesis, Virginia Commonwealth University).

Ali, L. I., Farhan, A. H., Elshafey, E., Elsayed, H. M., Badway, E. M. I. S., Al-Khatib, M. T. T., & Amin, H. M. (2025). Association between psychiatric symptoms, vitamin D serum level and parental stress in children and adolescents with epilepsy. *Zagazig University Medical Journal*, *31*(6), 2494-2508. https://doi.org/10.21608/zumj.2025.375846.3910

Al-Khotani, A., Gjelset, M., Naimi-Akbar, A., Hedenberg-Magnusson, B., Ernberg, M., & Christidis, N. (2018). Using the child behavior checklist to determine associations between psychosocial aspects and TMD-related pain in children and adolescents. *The Journal of Headache and Pain*, *19*(1), 88. https://doi.org/10.1186/s10194-018-0915-6

Baggio, M., Toffoli, L., Da Rold, M., Duma, G. M., Mento, G., Morao, V., Danieli, A., & Bonanni, P. (2022). Neuropsychological and behavioral profiles of self-limited epileptic syndromes of childhood: A cross-syndrome comparison. *Child Neuropsychology*, *28*(7), 878–902. https://doi.org/10.1080/09297049.2022.2028754

Bakri, M. H., Ismail, E. A., Elsedfy, G. O., Amr, M. A., & Ibrahim, A. (2014). Behavioral impact of sickle cell disease in young children with repeated hospitalization. *Saudi Journal of Anaesthesia*, *8*(4), 504–509. https://doi.org/10.4103/1658-354X.140867

Balottin, U., Maffioletti, E., Antonaci, E., Mensi, M. M., & Chiappedi, M. (2015). Emotive and behavior problems in adolescents with chronic daily headache. *Journal of Headache & Pain*, *1*(1), 3.

Battistutta, S., Aliverti, R., Montico, M., Zin, R., & Carrozzi, M. (2009). Chronic tension-type headache in adolescents. Clinical and psychological characteristics analyzed through self- and parent-report questionnaires. *Journal of Pediatric Psychology*, *34*(7), 697– 706. https://doi.org/10.1093/jpepsy/jsn102

Bender, H.A., Auciello, D., Morrison, C. E., MacAllister, W. S., & Zaroff, C. M. (2008). Comparing the convergent validity and clinical utility of the Behavior Assessment System for Children-Parent Rating Scales and Child Behavior Checklist in children with epilepsy. *Epilepsy & Behavior*, *13*(1), 237–242. https://doi.org/10.1016/ j.yebeh.2008.03.007

Bemis, H., Yarboi, J., Gerhardt, C. A., Vannatta, K., Desjardins, L., Murphy, L. K., ... & Compas, B. E. (2015). Childhood cancer in context: Sociodemographic factors, stress, and psychological distress among mothers and children. *Journal of Pediatric Psychology*, *40*(8), 733-743. https://doi.org/10.1093/jpepsy/jsv024

Berg, A. T., Vickrey, B. G., Testa, F. M., Levy, S. R., Shinnar, S., & DiMario, F. (2007). Behavior and social competency in idiopathic and cryptogenic childhood epilepsy. *Developmental Medicine & Child Neurology*, *49*(7), 487–492. https://doi.org/10.1111/ j.1469-8749.2007.00487.x

Bisacchi, N., Bal, M. O., Nardi, L., Bettocchi, I., D'Addabbo, G., Conti, V., ... & Cassio, A. (2011). Psychological and behavioural aspects in children and adolescents with congenital hypothyroidism diagnosed by neonatal screening: comparison between parents' and children's perceptions. *European Journal of Endocrinology*, *164*(2), 269-276. https://doi.org/10.1530/EJE-10-0761

Boscolo, V. (2017). Caratteristiche del funzionamento cognitivo e psicologico di soggetti preadolescenti e adolescenti con infezione da HIV Caratteristiche del funzionamento cognitivo e psicologico di soggetti preadolescenti e adolescenti con infezione da HIV Caratteristiche del funzionamento cognitivo e psicologico di soggetti preadolescenti 1 da trasmissione verticale [Characteristics of cognitive and psychological functioning in preadolescents and adolescents with HIV-1 infection acquired through vertical transmission] (Unpublished doctoral dissertation, University of Padova).

Brandsma, F. L., Spruijt, M. S., Rijken, M., Tan, R. N. G. B., Oepkes, D., Lopriore, E., & Van Klink, J. M. M. (2020). Behavioural outcome in twin–twin transfusion syndrome survivors treated with laser surgery. *Archives of Disease in Childhood - Fetal and Neonatal Edition*, *105*(3), 304–309. https://doi.org/10.1136/archdischild-2019-317080

Brumbaugh, J. E., Bell, E. F., Hirsch, S. C., Crenshaw, E. G., DeMauro, S. B., Adams-Chapman, I. S., ... & Eunice Kennedy Shriver National Institute of Child Health and Human Development Neonatal Research Network. (2023). Relationships between retinopathy of prematurity without ophthalmologic intervention and neurodevelopment and vision at 2 years. *Pediatric Research*, *94*(5), 1720-1730. https://doi.org/ 10.1038/s41390-021-01778-y

Chang, H. Y., Seo, J.-H., Kim, H. Y., Kwon, J.-W., Kim, B.-J., Kim, H. B., … Hong, S.-J. (2013). Allergic diseases in preschoolers are associated with psychological and behavioural problems. *Allergy, Asthma & Immunology Research*, *5*(5), 315-321. https://doi.org/10.4168/aair.2013.5.5.315

Chiou, S. S., Lin, P. C., Liao, Y. M., & Yang, P. (2019). A cross‐sectional follow‐up study of physical morbidities, neurocognitive function, and attention problems in post‐treatment childhood acute lymphoblastic leukemia survivors. *The Kaohsiung Journal of Medical Sciences*, *35*(6), 373-378. https://doi.org/10.1002/kjm2.12061

Chira, M., Ciotlaus, D. F., Vasile, O., Tinica, G., & Molnar, A. (2018). Behavioral development is better after early repair of tetralogy of Fallot. *Revista de Cercetare si Interventie Sociala*, *61*, 256-266.

Chisholm, A. K., Haebich, K. M., Pride, N. A., Walsh, K. S., Lami, F., Ure, A., … Payne, J. M. (2022). Delineating the autistic phenotype in children with neurofibromatosis type 1. *Molecular Autism*, *13*(1), 3. https://doi.org/10.1186/s13229-021-00481-3

Choi, E., Lee, M. J., Yun, H. J., Kim, J. H., Lee, J. S., Park, J. H., & Jung, J. A. (2016). Behavioral characteristics and parental stress in elementary school children with bronchial asthma and allergic rhinitis [in Korean]. *Allergy, Asthma & Respiratory Disease*, *4*(3), 205-211. http://dx.doi.org/10.4168/aard.2016.4.3.205

Compas, B. E., Desjardins, L., Vannatta, K., Young-Saleme, T., Rodriguez, E. M., Dunn, M., ... & Gerhardt, C. A. (2014). Children and adolescents coping with cancer: Self-and parent reports of coping and anxiety/depression. *Health Psychology*, *33*(8), 853-861. https://doi.org/10.1037/hea0000083

Crerand, C. E., Conrad, A. L., Bellucci, C. C., Albert, M., Heppner, C. E., Sheikh, F., ... & Kapp-Simon, K. A. (2024). Psychosocial outcomes in children with cleft lip and/or palate: associations of demographic, cleft morphologic, and treatment-related variables. *The Cleft Palate Craniofacial Journal*, *61*(11), 1784-1802. https://doi.org/ 10.1177/10556656231181581

de Jong, N. B., Elzinga-Plomp, A., Hulzebos, E. H., Poppe, R., Nijhof, S. L., & van Geelen, S. (2020). Coping with paediatric illness: Child’s play? Exploring the effectiveness of a play-and sports-based cognitive behavioural programme for children with chronic health conditions. *Clinical Child Psychology and Psychiatry*, *25*(3), 565-578. https://doi.org/10.1177/1359104520918327

Dollman, A. K., Figaji, A. A., & Schrieff-Elson, L. E. (2017). Academic and behavioral outcomes in school-age South African children following severe traumatic brain injury. *Frontiers in Neuroanatomy*, *11*, 121. https://doi.org/10.3389/fnana.2017.00121

Duchoslav, R.L. (2012). *The effects of pediatric acute lymphoblastic leukemia on social functioning: An investigation into the first year of treatment* (Unpublished doctoral dissertation, Utah State University).

Dufton, L. M., Dunn, M. J., & Compas, B. E. (2009). Anxiety and somatic complaints in children with recurrent abdominal pain and anxiety disorders. *Journal of Pediatric Psychology*, *34*(2), 176-186. https://doi.org/10.1093/jpepsy/jsn064

Eiffener, E., Eli, K., Ek, A., Sandvik, P., Somaraki, M., Kremers, S., ... & Nowicka, P. (2019). The influence of preschoolers' emotional and behavioural problems on obesity treatment outcomes: Secondary findings from a randomized controlled trial. *Pediatric Obesity*, *14*(11), e12556. https://doi.org/10.1111/ijpo.12556

Familiar, I., Ruisenor-Escudero, H., Giordani, B., Bangirana, P., Nakasujja, N., Opoka, R., & Boivin, M. (2015). Use of the Behavior Rating Inventory of Executive Function and Child Behavior Checklist in Ugandan children with HIV or a history of severe malaria. *Journal of Developmental & Behavioral Pediatrics*, *36*(4), 277-284. https://doi.org/10.1097/DBP.0000000000000149

Ferro, M. A., Van Lieshout, R. J., Ohayon, J., & Scott, J. G. (2016a). Emotional and behavioral problems in adolescents and young adults with food allergy. *Allergy*, *71*(4), 532–540. https://doi.org/10.1111/all.12829 72

Ferro, M. A., Van Lieshout, R. J., Scott, J. G., Alati, R., Mamun, A. A., & Dingle, K. (2016b). Condition-specific associations of symptoms of depression and anxiety in adolescents and young adults with asthma and food allergy. *Journal of Asthma*, *53*(3), 282-288. https://doi.org/10.3109/02770903.2015.1104694

Firoozi, M. (2017). Cognitive, emotional, and behavioral problems of children with hemophilia. *Iranian Journal of Blood and Cancer*, *9*(3), 69-74.

Fredericks, E. M., Lopez, M. J., Magee, J. C., Shieck, V., & Opipari‐Arrigan, L. (2007). Psychological functioning, nonadherence and health outcomes after pediatric liver trans plantation. *American Journal of Transplantation*, *7*(8), 1974–1983. https://doi.org/10.1111/j.1600-6143.2007.01878.x

Gaynor, J. W., Nord, A. S., Wernovsky, G., Bernbaum, J., Solot, C. B., Burnham, N., … Gerdes, M. (2009). Apolipoprotein E genotype modifies the risk of behavior problems after infant cardiac surgery. *Pediatrics*, *124*(1), 241–250. https://doi.org/10.1542/peds.2008-2281

Geerdink, L. M., van der Mheen, M., Sarvaas, G. J. du M., Kuipers, I. M., Frerich, S., ter Heide, H., … & Utens, E. M. W. J. (2020). Health-related quality of life, emotional and behavioral problems in children and adolescents with Ebstein anomaly. *Congenital Heart Disease*, *15*(6), 457–472. https://doi.org/10.32604/CHD.2020.012994

Getsuwan, S., Chuthapisith, J., Treepongkaruna, S., Butsriphum, N., Prabpram, W., Charoen thanakit, C. … Lertudomphonwanit, C. (2021). Behavior problems and cognitive function in pediatric liver transplant recipients. *Transplantation Proceedings*, *53*(2), 649–655. https://doi.org/10.1016/j.transproceed.2020.10.041

Goodwin, R. D., Robinson, M., Sly, P. D., & Holt, P. G. (2017). Childhood atopy and mental health: A prospective, longitudinal investigation. *Psychological Medicine*, *47*(2), 317– 325. https://doi.org/10.1017/S0033291716001896

Goodwin, R. D., Robinson, M., Sly, P. D., McKeague, I. W., Susser, E. S., Zubrick, S. R., Stanley, F. J., & Mattes, E. (2013). Severity and persistence of asthma and mental health: A birth cohort study. *Psychological Medicine*, *43*(6), 1313–1322. https://doi.org/10.1017/S0033291712001754 73

Hecker, H. E. (2016). *The psychological problems of children with HIV/AIDS in Tshwane* (Unpublished master thesis, University of Pretoria). http://hdl.handle.net/2263/60364

Hernandez-Reif, M., Shor-Posner, G., Baez, J., Soto, S., Mendoza, R., Castillo, R., ... & Zhang, G. (2008). Dominican children with HIV not receiving antiretrovirals: Massage therapy influences their behavior and development. *Evidence‐Based Complementary and Alternative Medicine*, *5*(3), 345-354. https://doi.org/10.1093/ecam/nem032

Hesdorffer, D. C., Caplan, R., & Berg, A. T. (2012). Familial clustering of epilepsy and behavioral disorders: Evidence for a shared genetic basis. *Epilepsia*, *53*(2), 301-307. https://doi.org/10.1111/j.1528-1167.2011.03351.x

Hostert, P. C. da C. P., Motta, A. B., & Enumo, S. R. F. (2015). Coping with hospitalization in children with cancer: The importance of the hospital school. *Estudos de Psicologia (Campinas)*, *32*, 627–639. https://doi.org/10.1590/0103-166X2015000400006

Jantarabenjakul, W. (2018). *Neurodevelopmental and neurobehavioral outcomes in early antiretroviral treated young children with perinatally-acquired HIV infection (PHIV) compared to age-matched perinatally HIV exposed uninfected children (PHEU)* (Unpublished doctoral dissertation, Chulalongkom University).

Johns, A. L., Wallace, E. R., Collett, B. R., Kapp-Simon, K. A., Drake, A. F., Heike, C. L., Kinter, S. L., Luquetti, D. V., Magee, L., Norton, S., Sie, K., & Speltz, M. L. (2021). Behavioral adjustment of preschool children with and without craniofacial microsomia. *The Cleft Palate Craniofacial Journal*, *58*(1), 42–53. https://doi.org/10.1177/ 1055665620947987

Kapp-Simon, K. A., Collett, B. R., Barr-Schinzel, M. A., Cradock, M. M., Buono, L. A., Pietila, K. E., & Speltz, M. L. (2012). Behavioral adjustment of toddler and preschool-aged children with single-suture craniosynostosis. *Plastic and Reconstructive Surgery*, *130*(3), 635-647. https://doi.org/10.1097/PRS.0b013e31825dc18b

Karver, C. L., Wade, S. L., Cassedy, A., Taylor, H. G., Stancin, T., Yeates, K. O., & Walz, N. C. (2012). Age at injury and long-term behavior problems after traumatic brain injury in young children. *Rehabilitation Psychology*, *57*(3), 256. https://doi.org/10.1037/ a0029522

Keenan, H. T., Clark, A. E., Holubkov, R., Cox, C. S., & Ewing-Cobbs, L. (2018). Psychosocial and executive function recovery trajectories one year after pediatric traumatic brain injury: the influence of age and injury severity. *Journal of Neurotrauma*, *35*(2), 286-296. https://doi.org/10.1089/neu.2017.5265

Kelmanson, I. A. (2018). Sleep disturbances and behavioural and emotional problems in preschool children with bilateral cerebral palsy. *Somnologie*, *22*(1), 18–26. https://doi.org/10.1007/s11818-017-0121-9

Lande, M. B., Adams, H., Falkner, B., Waldstein, S. R., Schwartz, G. J., Szilagyi, P. G., ... & Palumbo, D. (2009). Parental assessments of internalizing and externalizing behavior and executive function in children with primary hypertension. *The Journal of Pediatrics*, *154*(2), 207-212.https://doi.org/10.1016/j.jpeds.2008.08.017

Lepage, C., Tremblay, L., Bernier, A., Simard, M. N., Matte-Gagné, C., & Gallagher, A. (2025). Trajectories of behavioral and emotional problems in preschoolers with congenital heart disease. *Journal of Pediatric Psychology*, *50*(9), 897-909. https://doi.org/10.1093/ jpepsy/jsaf057

Lommel, K., Kapoor, S., Bamford, J., Melguizo, M. S., & Martinet, C. (2009). Juvenile primary fibromyalgia syndrome in an inpatient adolescent psychiatric population. *International Journal of Adolescent Medicine and Health*, *21*(4), 571-579. https://doi.org/10.1515/ ijamh.2009.21.4.571

Loutfi, K. S., Carvalho, A. M., Lamounier, J. A., & de Almeida Nascimento, J. (2011). ADHD and epilepsy: contributions from the use of behavioral rating scales to investigate psychiatric comorbidities. *Epilepsy & Behavior*, *20*(3), 484-489. https://doi.org/ 10.1016/j.yebeh.2010.12.043

Louw, K.-A., Ipser, J., Phillips, N., & Hoare, J. (2016). Correlates of emotional and behavioural problems in children with perinatally acquired HIV in Cape Town, South Africa. *AIDS Care*, *28*(7), 842–850. https://doi.org/10.1080/09540121.2016.1140892 74

Malucelli, G. S., Mercante, A., Pizza, F., Brunetti, V., Biscarini, F., Vandi, S., Mazzoni, A., Franceschini, C., Della Marca, G., Vollono, C., Chieffo, D. P. R., & Plazzi, G. (2024). Exploring the emotional and behavioural profile in paediatric narcolepsy type 1: A case–control study. *Journal of Sleep Research*, *33*(4), e14064. https://doi.org/ 10.1111/jsr.14064

Mandell, J. E., Howell, K. H., & Thurston, I. B. (2022). Developmental assets, defiance, and caregiver communication among black adolescents with high body weights. *Journal of Child and Family Studies*, *31*(4), 910-922. https://doi.org/10.1007/s10826-021-02043-w

Mavrea, K., Katsibardi, K., Roka, K., Pons, R., Efthymiou, V., Antoniou, A. S., ... & Bacopoulou, F. (2026). Cognitive and psychosocial burden of childhood cancer survivors in Greece: A case–control study. *Medical Sciences*, *14*(2), 171. https://doi.org/10.3390/medsci14020171

Maxwell, E. C., Ballantyne, T., Carlson, K. E., Hollatz, A. L., Clevenger, K. C., Armstrong-Wells, J., ... & Boada, R. (2017). Psychological outcomes after childhood arterial ischemic stroke. *Stroke*, *48*(suppl_1), ATP405-ATP405. https://doi.org/10.1161/ str.48.suppl_1.tp405

Meldrum, S. J., D’Vaz, N., Dunstan, J. A., Mori, T. A., Hird, K., Simmer, K., & Prescott, S. L. (2012). Allergic disease in the first year of life is associated with differences in subsequent neurodevelopment and behaviour. *Early Human Development*, *88*(7), 567– 573. https://doi.org/10.1016/j.earlhumdev.2011.12.032

Mendizabal, S. (2016). *La mémoire de travail visuelle chez l'enfant avec une épilepsie bénigne à pointes centro-temporales et chez l'enfant sain* [Visual working memory in children with benign centrotemporal spike epilepsy and in healthy children] (Unpublished doctoral dissertation, University of Montreal).

Miller, K. S., Vannatta, K., Compas, B. E., Vasey, M., McGoron, K. D., Salley, C. G., & Gerhardt, C. A. (2009). The role of coping and temperament in the adjustment of children with cancer. *Journal of Pediatric Psychology*, *34*(10), 1135–1143. https://doi.org/10.1093/jpepsy/jsp037

Moraes, M. M., Vaz, F. P. C., Roque, R. M. S. D. A., Mallozi, M. D. C., Solé, D., & Wandalsen, G. F. (2024). Behavioral disorders in children and adolescents with atopic dermatitis. *Jornal de Pediatria*, *100*(1), 93–99. https://doi.org/10.1016/j.jped.2023.08.006

Morava, E., Gardeitchik, T., Kozicz, T., De Boer, L., Koene, S., De Vries, M. C., ... & Verhaak, C. M. (2010). Depressive behaviour in children diagnosed with a mitochondrial disorder. *Mitochondrion*, *10*(5), 528-533. https://doi.org/10.1016/j.mito.2010.05.011

Moreira, F. D. S., Lima, A. B. D., Fonseca, P. C., & Maia Filho, H. D. S. (2014). Mental health of children and adolescents with epilepsy: Analysis of clinical and neuropsychological aspects. *Arquivos de Neuro-Psiquiatria*, *72*(8), 613–618. https://doi.org/10.1590/0004-282X20140098

Nagata, J. M., Hagan, M. J., Heyman, M. B., & Wojcicki, J. M. (2015). No association between obesity and behavior in low-income, preschool Latino children. *Journal of Health Care for the Poor and Underserved*, *26*(2), 410-420. https://doi.org/10.1353/hpu.2015.0042

Nardi, L., Zucchini, S., D’Alberton, F., Salardi, S., Maltoni, G., Bisacchi, N., Elleri, D., & Cicognani, A. (2008). Quality of life, psychological adjustment and metabolic control in youths with type 1 diabetes: A study with self- and parent-report questionnaires. *Pediatric Diabetes*, *9*(5), 496–503. https://doi.org/10.1111/j.1399-5448.2008.00414.x

Nazari, S., Koupaei, M. T. S., Shafiee, A., Kashani, Z. H. G., Bahraminia, E., Ansari, M., & Alipour, A. (2014). Emotional/behavioral problems in children with acute lymphoblastic leukemia: A case-control study. *International Journal of Hematology-Oncology and Stem Cell Research, 8*(2), 14-20.

Neto, E. V., de Souza Maia Filho, H., Monteiro, C. B., de Mattos Carvalho, L., da Cruz, T. S., de Barros, B. V., & Ribeiro, M. G. (2018). Behavioral and emotional problems in early-treated Brazilian children and adolescents with phenylketonuria. *Medical Science Monitor: International Medical Journal of Experimental and Clinical Research*, *24*, 7759–7769. https://doi.org/10.12659/MSM.909146

Nita, S. A., Teleanu, R. I., & Bajenaru, O. A. (2020). Psychological evaluation in childhood migraine—steps towards a psychological profile of the child migraineur. *Romanian Journal of Neurology*, *19*(1), 21-26. https://doi.org/10.37897/RJN.2020.1.3

Nunes, S., Argollo, N., Mota, M., Vieira, C., & de Sena, E. P. (2017). Comprehensive neuropsychological evaluation of children and adolescents with sickle cell anemia: a hospital-based sample. *Revista Brasileira de Hematologia e Hemoterapia*, *39*(1), 32-39. https://doi.org/10.1016/j.bjhh.2016.09.004

Operto, F. F., Pastorino, G. M. G., Pippa, F., Padovano, C., Vivenzio, V., Scuoppo, C., Pistola, I., & Coppola, G. (2021). Psychiatric symptoms and parental stress in children and adolescents with epilepsy. *Frontiers in Neurology*, *12,* 778410. https://doi.org/10.3389/ fneur.2021.778410

Palmieri, R., Albano, V., Guerriero, S., Craig, F., Torre, F. L., Filoni, S., Sardella, D., Petruzzelli, M. G., Lecce, P., & Giacomo, A. D. (2024). Beyond diagnosis: Preliminary study of impact on children and parents in neurodevelopmental disorders and juvenile idiopathic arthritis-associated uveitis. *Diagnostics*, *14*(3), 275. https://doi.org/10.3390/ diagnostics14030275

Park, M., Oh, S. H., Chang, S. O., Kim, C. S., & Lee, J. H. (2016). Long-term functional and behavioral–emotional outcomes in children with early cochlear implants: Parental testimonies. *International Journal of Pediatric Otorhinolaryngology*, *83*, 137-142. https://doi.org/10.1016/j.ijporl.2016.01.038

Park, S. H., Lee, J. S., Kim, Y. H., Byeon, J. H., Yoon, W., & Yoo, Y. (2023). Behavioral problems in Korean children and adolescents with atopic dermatitis. *Allergy, Asthma & Respiratory Disease*, *11*(3), 144-150. https://doi.org/10.4168/aard.2023.11.3.144

Parmeggiani, A., Boiani, F., Capponi, S., Duca, M., Angotti, M., Pignataro, V., ... & Franzoni, E. (2018). Neuropsychological profile in Italian children with neurofibromatosis type 1 (NF1) and their relationships with neuroradiological data: Preliminary results. *European Journal of Paediatric Neurology*, *22*(5), 822-830. https://doi.org/ 10.1016/j.ejpn.2018.04.016

Perez, K. M., Lee, E. B., Kahanda, S., Duis, J., Reyes, M., Jüppner, H., & Shoemaker, A. H. (2018). Cognitive and behavioral phenotype of children with pseudohypoparathyroidism type 1A. *American Journal of Medical Genetics. Part A*, *176*(2), 283–289. https://doi.org/10.1002/ajmg.a.38534

Peterson, R. L., Kirkwood, M. W., Taylor, H. G., Stancin, T., Brown, T. M., & Wade, S. L. (2013). Adolescents’ internalizing problems following traumatic brain injury are related to parents’ psychiatric symptoms. *The Journal of Head Trauma Rehabilitation*, *28*(5), E1-E12. https://doi.org/10.1097/HTR.0b013e318263f5ba

Pound, C. M., Clark, C., Ni, A., Athale, U., Lewis, V., & Halton, J. M. (2012). Corticosteroids, behavior, and quality of life in children treated for acute lymphoblastic leukemia; a multicentered trial. *Journal of Pediatric Hematology/Oncology*, *34*(7), 517-523. https://doi.org/10.1097/MPH.0b013e318257fdac

Pride, N. A., Haebich, K. M., Walsh, K. S., Lami, F., Rouel, M., Maier, A., Chisholm, A. K., Lorenzo, J., Hearps, S. J. C., North, K. N., Payne, J. M. (2023). Sensory processing in children and adolescents with neurofibromatosis type 1. *Cancers*, *15*(14), 3612. https://doi.org/10.3390/cancers15143612

Prussien, K. V., DeBaun, M. R., Yarboi, J., Bemis, H., McNally, C., Williams, E., & Compas, B. E. (2018). Cognitive function, coping, and depressive symptoms in children and adolescents with sickle cell disease. *Journal of Pediatric Psychology*, *43*(5), 543–551. https://doi.org/10.1093/jpepsy/jsx141

Rackauskaite, G., Bilenberg, N., Bech, B. H., Uldall, P., & Østergaard, J. R. (2016). Screening for psychopathology in a national cohort of 8- to 15-year-old children with cerebral palsy. *Research in Developmental Disabilities*, *49–50*, 171–180. https://doi.org/10.1016/j.ridd.2015.11.019

Rocca, F. L., Finotti, E., Pizza, F., Ingravallo, F., Gatta, M., Bruni, O., & Plazzi, G. (2016). Psychosocial profile and quality of life in children with type 1 narcolepsy: A case-control study. *Sleep*, *39*(7), 1389–1398. https://doi.org/10.5665/sleep.5970

Roessler, H., van der Heuvel, L., Shields, K., Guilliams, K., Knoers, N., van Haaften, G., Grange, D., & van Haelst, M. (2021). Behavioral and cognitive functioning in individuals with Cantu syndrome. *American Journal of Medical Genetics Part A*, *185*(8), 2434–2444. https://doi.org/10.1002/ajmg.a.62348

Rofey, D. L., Zeller, M. H., Brode, C., Reiter‐Purtill, J., Mikhail, C., Washington, G., ... & TeenView Writing Group in cooperation with the Teen‐LABS Consortium. (2015). A multisite view of psychosocial risks in patients presenting for bariatric surgery. *Obesity*, *23*(6), 1218-1225. https://doi.org/10.1002/oby.21065

Sady, M. D., Coburn, S. S., Kramer, Z., Streisand, R., & Kahn, I. (2025). Associations between executive functioning and adherence in pediatric celiac disease. *Children’s Health Care: Journal of the Association for the Children‘s Health Care*, *54*(3), 296– 310. https://doi.org/10.1080/02739615.2023.2259291

Saini, M.K. (2023). *Cognitive, language, and psychosocial outcomes in children with complex congenital heart disease: A longitudinal cohort study from infancy to early school years* (Unpublished doctoral dissertation, UCL Great Ormond Street Institute of Child Health).

Sarrechia, I., De Wolf, D., Miatton, M., François, K., Gewillig, M., Meyns, B., & Vingerhoets, G. (2015). Neurodevelopment and behavior after transcatheter versus surgical closure of secundum type a trial septal defect. *The Journal of Pediatrics*, *166*(1), 31–38.e1. https://doi.org/10.1016/j.jpeds.2014.08.039

Shinnar, R. C., Shinnar, S., Cnaan, A., Clark, P., Dlugos, D., Hirtz, D. G., Hu, F., Liu, C., Masur, D., Weiss, E. F., & Glauser, T. A. (2017). Pretreatment behavior and subsequent medication effects in childhood absence epilepsy. *Neurology*, *89*(16), 1698–1706. https://doi.org/10.1212/WNL.0000000000004514 77

Singh, H., Aneja, S., Unni, K. E. S., Seth, A., & Kumar, V. (2012). A study of educational underachievement in Indian children with epilepsy. *Brain and Development*, *34*(6), 504-510. https://doi.org/10.1016/j.braindev.2011.08.011

Skokauskas, N., Sweeny, E., Meehan, J., & Gallagher, L. (2012). Mental health problems in children with Prader Willi syndrome. *Journal of the Canadian Academy of Child and Adolescent Psychiatry = Journal De l’Academie Canadienne De Psychiatrie De L’en fant Et De L’adolescent*, *21*(3), 194–203.

Soedjana, H., Bangun, K., & Christine, S. (2022). Evaluating psychosocial problems in school-age children with cleft lip and palate in Bandung, Indonesia using CBCL/6-18. *The Cleft Palate Craniofacial Journal*, *59*(10), 1246-1252. https://doi.org/10.1177/ 10556656211040703

Stavropoulos, I., Pervanidou, P., Gnardellis, C., Loli, N., Theodorou, V., Mantzou, A., Soukou, F., Sinani, O., & Chrousos, G. P. (2017). Increased hair cortisol and antecedent somatic complaints in children with a first epileptic seizure. *Epilepsy & Behavior*, *68*, 146–152. https://doi.org/10.1016/j.yebeh.2016.12.015

Taddei, M., Erbetta, A., Esposito, S., Saletti, V., Bulgheroni, S., & Riva, D. (2019). Brain tumors in NF1 children: Influence on neurocognitive and behavioral outcome. *Cancers*, *11*(11), 1772. https://doi.org/10.3390/cancers11111772

Tester, M. A., Riehm, K. E., Perry, F., Franciosi, S., Escudero, C. A., Maghrabi, K., Sneddon, P., & Sanatani, S. (2021). Paediatric supraventricular tachycardia patients potentially more at risk of developing psychological difficulties compared to healthy peers. *Acta Paediatrica*, *110*(3), 1017–1024. https://doi.org/10.1111/apa.15556

Tremolada, M., Bonichini, S., Piovan, V., Pastore, N., & Mazzoldi, M. (2016). QoL in Southtyrol T1D pediatric patients: Psychosocial well-being, quality of life, health status and family conflicts in a Southtyrolean group of pediatric patients with diabetes mellitus type I. *International Neuropsychiatric Disease Journal*, *8*(1), 1–17. https://doi.org/10.9734/INDJ/2016/28553

Uhlig, S.C. (2023). *Auswirkungen der Behandlung mit der Tübinger Spornplatte bei Kindern mit Robin Sequenz auf das Auftreten eines obstruktiven Schlafapnoesyndroms und kognitive Fähigkeiten im Schulalter* [Effects of treatment with the Tübingen Spur Plate in children with Robin sequence on the incidence of obstructive sleep apnea syndrome and cognitive abilities during school age] (Unpublished doctoral dissertation, Eberhard Karls Universität zu Tübingen).

Usubini, A. G., Marazzi, N., Abbruzzese, L., Tringali, G., De Micheli, R., Castelnuovo, G., & Sartorio, A. (2025). Emotional and behavioural adjustment in children and adolescents with short stature vs. Their normal-statured peers. *Journal of Clinical Medicine*, *14*(2), 538. https://doi.org/10.3390/jcm14020538

Usubini, A. G., Bottacchi, M., Bondesan, A., Marazzi, N., Castelnuovo, G., & Sartorio, A. (2024). Behavioral and emotional problems in children and adolescents with obesity: A preliminary report. *Journal of Clinical Medicine*, *13*(2), 459. https://doi.org/10.3390/ jcm13020459

van der Mheen, M., van der Meulen, M. H., den Boer, S. L., Schreutelkamp, D. J., van der Ende, J., de Nijs, P. F. A., … Dalinghaus, M. (2020). Emotional and behavioral problems in children with dilated cardiomyopathy. *European Journal of Cardiovascular Nursing*, *19*(4), 291–300. https://doi.org/10.1177/1474515119876148

van der Plas, E., Schachar, R. J., Hitzler, J., Crosbie, J., Guger, S. L., Spiegler, B. J., Ito, S., & Nieman, B. J. (2017). Brain structure, working memory and response inhibition in childhood leukemia survivors. *Brain and Behavior*, *7*(2), e00621. https://doi.org/ 10.1002/brb3.621

Van Vliet, K., van Ginkel, W. G., Jahja, R., Daly, A., MacDonald, A., De Laet, C., ... & van Spronsen, F. J. (2019). Emotional and behavioral problems, quality of life and metabolic control in NTBC-treated Tyrosinemia type 1 patients. *Orphanet Journal of Rare Diseases*, *14*(1), 285. https://doi.org/10.1186/s13023-019-1259-2

Vaz, F. P. C., Braghiroli, M. M. M., Nogueira, P. C. K., Solé, D., & Wandalsen, G. F. (2026). Emotional and behavioral problems in children and adolescents with asthma and their association with disease severity. *Pediatric Pulmonology*, *61*(5), e71672. https://doi.org/10.1002/ppul.71672

Visser, M. J., Hecker, H. E., & Jordaan, J. (2018). A comparative study of the psychological problems of HIV-infected and HIV-uninfected children in a South African sample. *AIDS Care*, *30*(5), 596–603. https://doi.org/10.1080/09540121.2017.1417530

Vourdoumpa, A., Paltoglou, G., Manou, M., Koutaki, D., Kassari, P., Papadopoulou, M., ... & Charmandari, E. (2026). Parallel improvement of both mental and cardiometabolic health in children and adolescents with overweight and obesity, following the implementation of a multidisciplinary lifestyle intervention program. *Nutrients*, *18*(1), 150. https://doi.org/10.3390/nu18010150

Wade, S. L., Kaizar, E. E., Narad, M. E., Zang, H., Kurowski, B. G., Miley, A. E., ... & Zhang, N. (2020). Behavior problems following childhood TBI: The role of sex, age, and time since injury. *The Journal of Head Trauma Rehabilitation*, *35*(5), E393-E404. https://doi.org/10.1097/HTR.0000000000000567

Wahab, R. J., Beth, S. A., Derks, I. P., Jansen, P. W., Moll, H. A., & Kiefte-de Jong, J. C. (2019). Celiac disease autoimmunity and emotional and behavioral problems in childhood. *Pediatrics*, *144*(4), e20183933. https://doi.org/10.1542/peds.2018-3933

Wolock, E. R., Queen, A. H., Rodríguez, G. M., & Weisz, J. R. (2020). Chronic illness and internalizing symptomatology in a transdiagnostic clinical sample of youth. *Journal of Pediatric Psychology*, *45*(6), 633–642. https://doi.org/10.1093/jpepsy/jsaa028

Yahia, S., Elwasify, M., Ghozzy, D., El-Menayyar, S. M., Seleem, M. A., & Abdelmoneim, Z. (2026). Emotional and behavioral problems in children and adolescents with neurofibromatosis type 1. *Pediatric Research*, 1-7. https://doi.org/10.1038/s41390-026-05116-y

Yumul, J. N., McKinlay, A., Anderson, V., & Catroppa, C. (2024). Behaviour outcomes three months after mild TBI in preschool children. *Neuropsychological Rehabilitation*, *34*(4), 600-618. https://doi.org/10.1080/09602011.2023.2224030

Zambrelli, E., Turner, K., Peron, A., Leidi, A., La Briola, F., Vignoli, A., & Canevini, M. P. (2021). Sleep and behavior in children and adolescents with tuberous sclerosis complex. *American Journal of Medical Genetics Part A*, *185*(5), 1421–1429. https://doi.org/ 10.1002/ajmg.a.62123

Zambrelli, E., Turner, K., Vignoli, A., La Briola, F., Dionisio, S., Malanchini, S., Galli, F., & Canevini, M. P. (2020). Sleep disturbances in Italian children and adolescents with epilepsy: A questionnaire study. *Epilepsy & Behavior*, *106*, 107014. https://doi.org/10.1016/j.yebeh.2020.107014

Zanaboni, M. P., Raspanti, A., Quaranta, C. A., Bova, M. S., Celario, M., Mensi, M. M., Milito, G., Operto, F. F., Pasca, L., Pastorino, G. M. G., Raviglione, F., Vedana, M., Vignoli, A., & De Giorgis, V. (2026). Behind the seizures: Exploring the emotional burden of adolescents with idiopathic generalized epilepsy. *Epilepsy & Behavior*, 110912. https://doi.org/10.1016/j.yebeh.2026.110912

Zanato, S., Traverso, A., Tremolada, M., Sinatora, F., Porreca, A., Pozziani, G., Di Florio, N., Capello, F., Marzollo, A., Tumino, M., Cattelan, C., Basso, G., & Messina, C. (2017). Psychopathological aspects in childhood hematopoietic stem cell transplantation (HSCT): The perception of parents and adolescents. *Frontiers in Psychology*, *8*, 272. https://doi.org/10.3389/fpsyg.2017.00272

Zeller, M. H., Roehrig, H. R., Modi, A. C., Daniels, S. R., & Inge, T. H. (2006). Health-related quality of life and depressive symptoms in adolescents with extreme obesity presenting for bariatric surgery. *Pediatrics*, *117*(4), 1155–1161. https://doi.org/10.1542/peds.2005-1141

Zoair, A. M., Elgohary, T. M., Seleem, M. A., Elamrosy, D. M., Amer, S. F., & Saada, S. A. (2015). Psychosocial profile of a sample of Egyptian children with rheumatic heart disease. *Middle East Current Psychiatry*, *22*(2), 114. https://doi.org/10.1097/01.XME. 0000461678.30239.4f

**ESM 3: Funnel Plots**

**Affective Problems**

**Anxiety Problems**

**Somatic Problems**

**ADH Problems**

**Oppositional Defiant Problems**

**Conduct Problems**

**ESM 4: Condition-Specific Analyses**

|  | Atopic Conditions | | | | | | | Cancer | | | | | | |
| --- | --- | --- | --- | --- | --- | --- | --- | --- | --- | --- | --- | --- | --- | --- |
|  | *N* | *k* | *g* | *LL* | *UL* | *t* | *Q* | *N* | *k* | *g* | *LL* | *UL* | *t* | *Q* |
| Affective Problems | 12 | 22 | .394 | .204 | .583 | 4.31^c^ | 168.90^c^ | 9 | 10 | .706 | .538 | .874 | 8.80^c^ | 26.61 |
| Anxiety Problems | 12 | 22 | .401 | .205 | .596 | 4.24^c^ | 163.31^c^ | 8 | 9 | .560 | .236 | .884 | 3.65^b^ | 53.58^c^ |
| Somatic Problems | 6 | 11 | .631 | .301 | .962 | 4.26^b^ | 46.12^c^ | 5 | 5 | .780 | .318 | 1.241 | 4.00^b^ | 18.32^a^ |
| ADH Problems | 9 | 19 | .280 | .151 | .410 | 4.53^c^ | 66.60^c^ | 6 | 7 | .608 | .123 | 1.093 | 2.79^a^ | 48.26^c^ |
| OD Problems | 9 | 19 | .142 | .042 | .242 | 2.96^b^ | 42.20^b^ | 4 | 5 | .375 | -.342 | 1.092 | 1.21 | 56.37^c^ |
| Conduct Problems | 8 | 15 | .196 | .039 | .353 | 2.66^a^ | 33.50^b^ | 4 | 4 | .660 | -.053 | 1.374 | 2.26 | 23.61^c^ |

|  | Cardiovascular Diseases | | | | | | | Chronic Pain | | | | | | |
| --- | --- | --- | --- | --- | --- | --- | --- | --- | --- | --- | --- | --- | --- | --- |
|  | *N* | *k* | *g* | *LL* | *UL* | *t* | *Q* | *N* | *k* | *g* | *LL* | *UL* | *t* | *Q* |
| Affective Problems | 11 | 12 | .458 | .287 | .629 | 5.68^c^ | 30.41^a^ | 5 | 5 | .782 | .307 | 1.263 | 4.24^b^ | 19.46^b^ |
| Anxiety Problems | 11 | 13 | .302 | .149 | .455 | 4.17^c^ | 29.69^a^ | 6 | 6 | .688 | .149 | 1.231 | 3.12^a^ | 30.48^c^ |
| Somatic Problems | 8 | 9 | .468 | .240 | .697 | 4.52^c^ | 18.59 | 5 | 5 | 1.005 | .458 | 1.462 | 5.65^b^ | 15.97^b^ |
| ADH Problems | 10 | 12 | .240 | .055 | .425 | 2.75^a^ | 51.07^c^ | 4 | 4 | .432 | -.166 | 1.030 | 2.30 | 14.03^c^ |
| OD Problems | 11 | 13 | .213 | -.038 | .464 | 1.79 | 44.92^c^ | 4 | 4 | .448 | -.113 | 1.010 | 2.54 | 10.93^a^ |
| Conduct Problems | 8 | 10 | .251 | -.013 | .514 | 2.00 | 56.33^c^ | 4 | 4 | .200 | -.081 | .480 | 2,27 | 4.42 |

|  | Epilepsy | | | | | | | HIV Infection/AIDS | | | | | | |
| --- | --- | --- | --- | --- | --- | --- | --- | --- | --- | --- | --- | --- | --- | --- |
|  | *N* | *k* | *g* | *LL* | *UL* | *t* | *Q* | *N* | *k* | *g* | *LL* | *UL* | *t* | *Q* |
| Affective Problems | 16 | 20 | .739 | .494 | .984 | 6.21^c^ | 132.77^c^ | 7 | 8 | .617 | .312 | .923 | 4.57^b^ | 46.71^c^ |
| Anxiety Problems | 14 | 17 | .661 | .450 | .872 | 6.60^c^ | 70.30^c^ | 7 | 8 | .271 | .015 | .527 | 2.40^a^ | 23.07^b^ |
| Somatic Problems | 13 | 15 | .512 | .314 | .710 | 5.50^c^ | 41.21^c^ | 5 | 5 | .611 | .142 | 1.079 | 3.35^a^ | 27.94^c^ |
| ADH Problems | 15 | 19 | .728 | .504 | .952 | 6.80^c^ | 134.97^c^ | 7 | 8 | .159 | -.144 | .463 | 1.19 | 29.96^c^ |
| OD Problems | 14 | 18 | .574 | .352 | .796 | 5.43^c^ | 104.32^c^ | 7 | 8 | .149 | -.166 | .463 | 1.07 | 35.03^c^ |
| Conduct Problems | 15 | 19 | .585 | .394 | .776 | 6.40^c^ | 96.35^c^ | 5 | 5 | .202 | -.339 | .744 | .96 | 39.85^c^ |

|  | Obesity | | | | | | | Traumatic Brain Injury | | | | | | |
| --- | --- | --- | --- | --- | --- | --- | --- | --- | --- | --- | --- | --- | --- | --- |
|  | *N* | *k* | *g* | *LL* | *UL* | *t* | *Q* | *N* | *k* | *g* | *LL* | *UL* | *t* | *Q* |
| Affective Problems | 10 | 7 | .743 | .341 | 1.145 | 4.18^b^ | 63.73^c^ | 5 | 11 | .746 | .648 | .843 | 16.25^c^ | 16.89 |
| Anxiety Problems | 9 | 6 | .543 | .139 | .947 | 3.10^a^ | 43.78^c^ | 6 | 13 | .595 | .496 | .694 | 12.63^c^ | 22.53 |
| Somatic Problems | 7 | 4 | .651 | .002 | 1.301 | 2.45^a^ | 61.10^c^ | 3 | 7 | .780 | .654 | .905 | 14.34^c^ | 5.18 |
| ADH Problems | 9 | 6 | .413 | .013 | .813 | 2.38^a^ | 52.27^c^ | 5 | 11 | .591 | .238 | .943 | 3.62^b^ | 21.84 |
| OD Problems | 11 | 7 | .432 | .165 | .699 | 3.60^b^ | 32.62^c^ | 2 | 5 | .871 | -.061 | 1.801 | 2.60 | 5.60 |
| Conduct Problems | 4 | 7 | .495 | .155 | .835 | 3.56^a^ | 19.50^b^ | 5 | 5 | .202 | .339 | .744 | .96 | 39.85^c^ |

*Note*. *N* = number of effect sizes, *k* = umber of samples, *g* = weighted mean effect size, LL/UL = lower/upper level of the 95%-confidence interval, *t* = test for significance of *g*, *Q* = test for heterogeneity, ADH = attention deficit hyperactivity, OD = oppositional defiant, ^a^ *p* < .05, ^b^ *p* < .01, ^c^ *p* < .001.
